# Structural basis of tankyrase activation by polymerization

**DOI:** 10.1038/s41586-022-05449-8

**Published:** 2022-11-23

**Authors:** Nisha Pillay, Laura Mariotti, Mariola Zaleska, Oviya Inian, Matthew Jessop, Sam Hibbs, Ambroise Desfosses, Paul C. R. Hopkins, Catherine M. Templeton, Fabienne Beuron, Edward P. Morris, Sebastian Guettler

**Affiliations:** 1 Division of Structural Biology, The Institute of Cancer Research (ICR), London, UK; 2 Division of Cancer Biology, The Institute of Cancer Research (ICR), London, UK; 3 Institut de Biologie Structurale (IBS), University Grenoble Alpes, CEA, CNRS, Grenoble, France

**Keywords:** Intracellular signalling peptides and proteins, Cryoelectron microscopy, Transferases, PolyADP-ribosylation

## Abstract

The poly-ADP-ribosyltransferase tankyrase (TNKS, TNKS2) controls a wide range of disease-relevant cellular processes, including WNT–β-catenin signalling, telomere length maintenance, Hippo signalling, DNA damage repair and glucose homeostasis^1,2^. This has incentivized the development of tankyrase inhibitors. Notwithstanding, our knowledge of the mechanisms that control tankyrase activity has remained limited. Both catalytic and non-catalytic functions of tankyrase depend on its filamentous polymerization^3–5^. Here we report the cryo-electron microscopy reconstruction of a filament formed by a minimal active unit of tankyrase, comprising the polymerizing sterile alpha motif (SAM) domain and its adjacent catalytic domain. The SAM domain forms a novel antiparallel double helix, positioning the protruding catalytic domains for recurring head-to-head and tail-to-tail interactions. The head interactions are highly conserved among tankyrases and induce an allosteric switch in the active site within the catalytic domain to promote catalysis. Although the tail interactions have a limited effect on catalysis, they are essential to tankyrase function in WNT–β-catenin signalling. This work reveals a novel SAM domain polymerization mode, illustrates how supramolecular assembly controls catalytic and non-catalytic functions, provides important structural insights into the regulation of a non-DNA-dependent poly-ADP-ribosyltransferase and will guide future efforts to modulate tankyrase and decipher its contribution to disease mechanisms.

## Abstract (web summary)

Structural insights into the poly-ADP-ribosyltransferase tankyrase reveal its filamentous architecture and illustrate how assembly controls catalytic and non-catalytic functions.

## Main

**Fig. 1:**
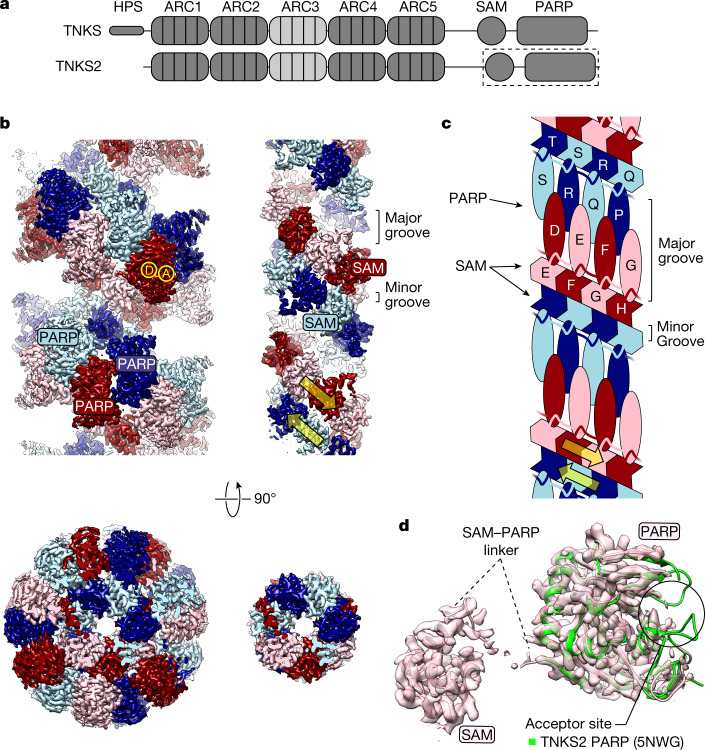
Architecture of the TNKS2 SAM–PARP filament. **a**, Domain organization of TNKS and TNKS2. **b**, Cryo-EM maps (before final sharpening in Phenix for model building) of TNKS2 SAM–PARP (left) and after masking out PARP (right). The antiparallel protofilaments are related to each other by D1 symmetry. A, acceptor site; D, donor site. Yellow arrows indicate protofilament polarity. **c**, Schematic representation of the quaternary filament structure, with letters indicating different protomers (see Extended Data Fig. 3j). **d**, Additionally sharpened cryo-EM map and model of a single TNKS2 SAM–PARP protomer. The PARP domain from PDB 5NWG^33^ is superimposed in green to illustrate poorly resolved features of the acceptor site. See Extended Data Fig. 2 for data processing details, and Extended Data Fig. 3 for map details and analysis of the G1032W^TNKS2^ mutation.

Poly(ADP-ribosyl)ation (PARylation) is a ubiquitous but understudied post-translational modification implicated in a wide range of biological activities^6^. The DNA-damage-induced poly-ADP-ribosyltransferases PARP1 and PARP2 are therapeutic targets in ovarian, breast, prostate and pancreatic cancers^7^. Although their regulation is fairly well understood, that of the other two PAR-producing family members, tankyrase 1 and tankyrase 2 (TNKS and TNKS2, respectively; Fig. 1a), is not^8,9^. Tankyrase-regulated processes include WNT–β-catenin signalling^10^, telomere length maintenance and cohesion^11^, Hippo signalling^12^, glucose metabolism^13^, mitosis^14^ and DNA repair^15^. These functions have incentivized the development of tankyrase inhibitors with potential therapeutic utility in cancer, neurodegeneration, diabetes and fibrosis^16^. Tankyrases assemble into helical filaments through their polymerizing SAM domain^3–5,17^. Polymerization of tankyrase facilitates (1) binding of substrates and (2) PARP catalytic activation^4,5,18^, and is essential for tankyrase function in WNT–β-catenin signalling, both through catalysis-dependent and catalysis-independent (scaffolding) mechanisms^4,5,18^.

How tankyrase polymerization induces its PARP activity has remained unknown. Here we describe the 3 Å cryo-electron microscopy (cryo-EM) structure of a polymeric minimal active unit of tankyrase, revealing a novel double-helical architecture that enables reciprocal interactions between PARP domains to allosterically activate tankyrase.

## Overall filament architecture

**Extended Data Fig. 1:**
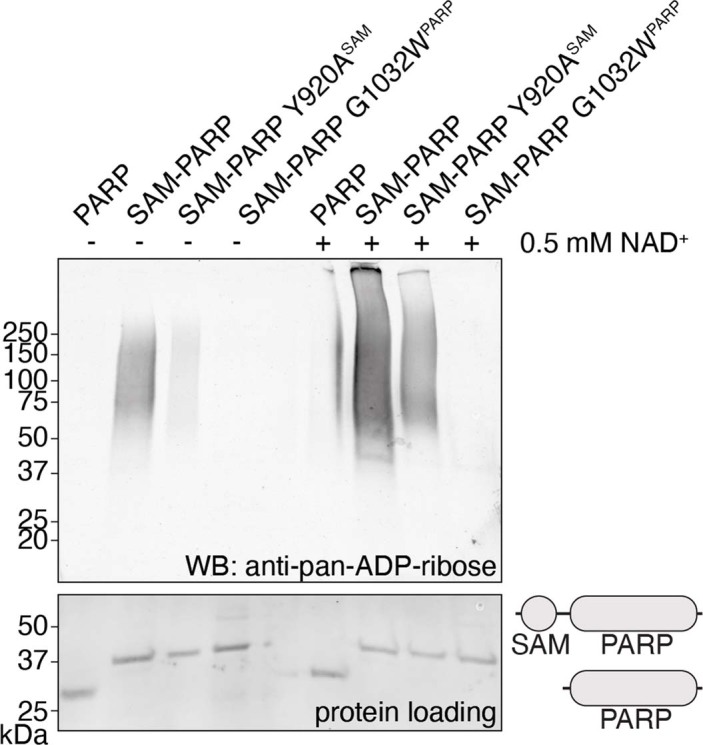
TNKS2 SAM-PARP polymerisation promotes auto-PARylation. Recombinant TNKS2 PARP and SAM-PARP were analysed for auto-PARylation by Western blotting, either before or after *in vitro* PARylation by incubation with NAD^+^. Y920A^SAM^, polymerisation-deficient mutant variant; G1032W^PARP^, catalytically inactive mutant variant. Top, anti-pan-ADP-ribose Western blot; bottom, Revert-stained membrane for protein load. One representative out of three independent experiments with similar results is shown.

**Extended Data Fig. 2:**
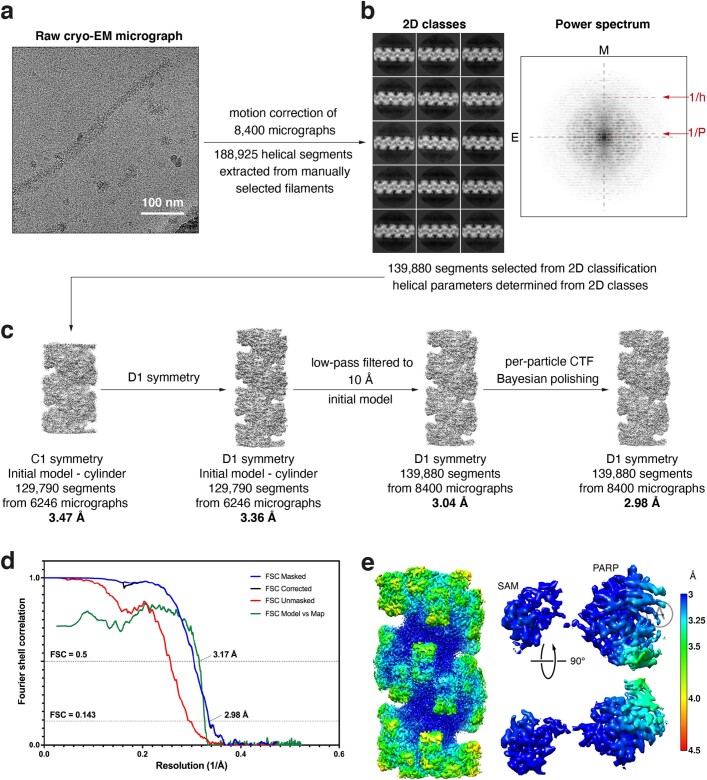
SAM-PARP cryo-EM data processing. **a**, Representative cryo-EM micrograph (of 8,400) showing SAM-PARP filaments; scale bar, 100 nm. **b**, Representative 2D class averages and average power spectrum from several class averages. M, meridian; E, equator. The first layer line from the equator (corresponding to the helical pitch P) and the first meridional layer line (corresponding to the helical rise h) are indicated. Particles from well-refined 2D classes were selected for subsequent processing. **c**, Workflow of 3D helical reconstruction. Refinements were iteratively improved by applying D1 symmetry, addition of particles and polishing steps. The numbers of segments contributing to each map are indicated. **d**, Gold-standard Fourier shell correlation (FSC) curves for the final half-maps. The estimated global resolution at 0.143 FSC is indicated. The FSC curves of the masked, unmasked and corrected maps are coloured in blue, red and black, respectively. The FSC curve for the model vs. map is shown in green. The estimated global model resolution at 0.5 FSC is indicated. **e**, Local resolution calculated by RELION. The surface colour of the map (for the filament and an individual, centrally located SAM-PARP protomer corresponding to chain E of the model) is rendered by local resolution, as shown in the colour scale. The acceptor site (A) is indicated. To facilitate model building, the map shown was further locally sharpened using Phenix Autosharpen. See Methods for data processing details.

**Extended Data Fig. 3:**
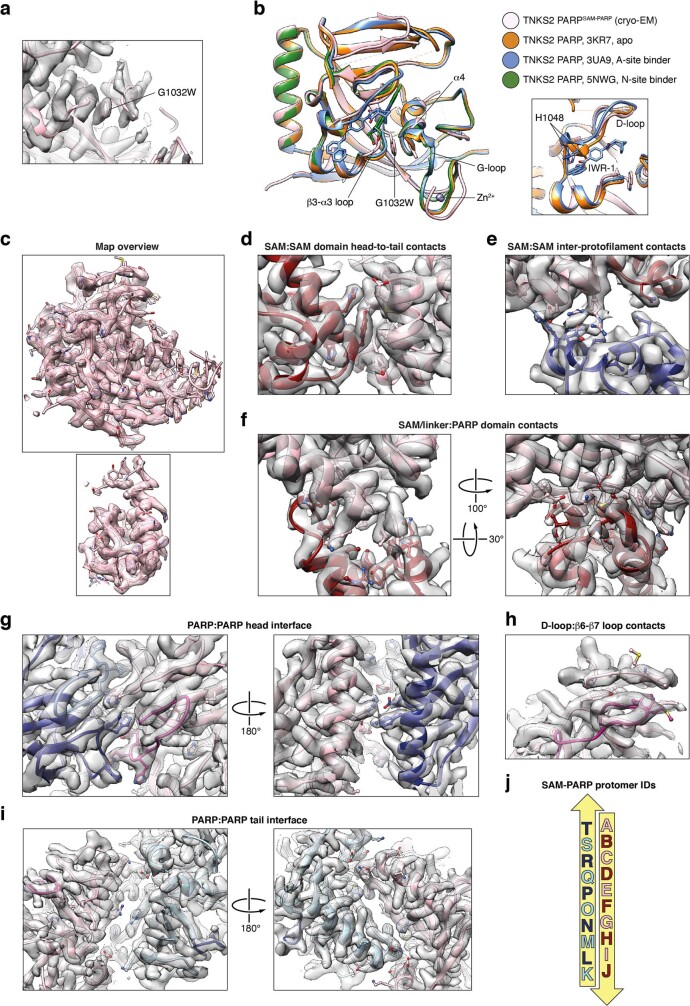
Cryo-EM target and map features. **a**, Cryo-EM map contoured at 8.73 and corresponding model of TNKS2 SAM-PARP chain E in cartoon representation with the G1032W^PARP^ mutant residue in stick representation. **b**, Superimposition of PARP domain from TNKS2 SAM-PARP G1032W^PARP^ (pink) with PARP domains from crystal structures: apo-TNKS2 PARP (3KR7, orange)^63^, TNKS2 PARP in complex with a small-molecule inhibitor binding the adenosine subsite (IWR-1, 3UA9, blue)^64^, TNKS2 PARP in complex with a small-molecule inhibitor binding the nicotinamide subsite (5NWG, green)^33^. The tryptophane introduced by the inactivating G1032W^PARP^ mutation displaces the β3-α3 loop and helix α4 plus the subsequent G-loop relative to each other, which likely accounts for catalytic inactivation^4,65^. It is unlikely that the disorder within the distant acceptor site is due to the mutation. However, it is possible that the mutation contributes to the apparent disorder in the G-loop and Zn^2+^-binding site. The region of the PARP domain affected by the G1032W^PARP^ mutation is situated on the filament periphery, distal to observed inter-domain contacts. The inset shows conformational differences of the D-loop base and H1048, which stacks with the adenine-mimetic amide region of the A-site binder IWR-1^64^. This stacking interaction also likely occurs with NAD^+^^66^. **c**, General overview of the TNKS2 SAM-PARP G1032W^PARP^ cryo-EM map (after Phenix Autosharpen) used for model building. The map is contoured at 8.73. The model is shown in cartoon representation with side chains in stick representation. **d-i**, Cryo-EM maps corresponding to structural representations shown in the main figures. Maps are contoured at 8.73, with the exception of e, which is contoured at 6.3 to better display the density for the two R896 conformers with lower individual occupancies. **j**, IDs of SAM-PARP protomers for the 20-protomer filament built into the cryo-EM map.

To understand how polymerization activates tankyrase, we determined the 3 Å cryo-EM structure of a minimal active TNKS2 SAM–PARP unit by helical reconstruction (Extended Data Fig. 1; see Methods, Extended Data Table 1 and Extended Data Figs. 2 and 3 for EM). To prevent auto-PARylation-induced depolymerization^3^, we used a catalytically inactive G1032W^PARP^-mutant variant^4^ (Extended Data Fig. 3b).

**Fig. 2:**
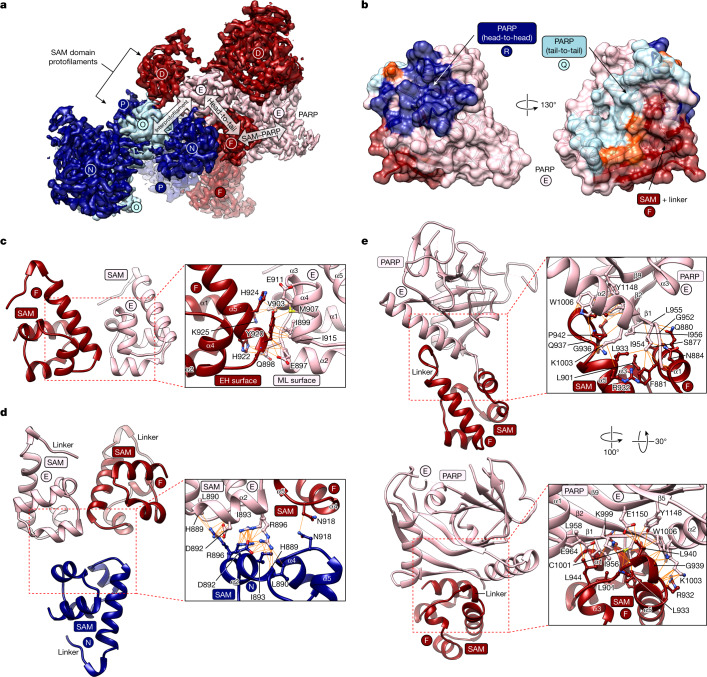
SAM domain contacts within the TNKS2 SAM–PARP filament. **a**, Cryo-EM map and model showing three adjacent SAM–PARP protomers each from antiparallel protofilaments (D, E, F and N, O, P). Arrows indicate domain interactions, and letters refer to different SAM–PARP protomers (see Fig. 1c). **b**, Model of the TNKS2 PARP domain with buried surfaces identified by PISA^21^ highlighted in the respective colour code of the interacting domain (see Fig. 1c). Residues buried by two adjacent surfaces are shown in orange. **c**, Cartoon representation of SAM–SAM head-to-tail contacts (323 Å^2^), with key interacting residues shown in stick representation and bonds indicated by orange lines. EH, end helix; ML, mid-loop. **d**, As for **c** but for SAM–SAM interprotofilament contacts (161 Å^2^). **e**, As for **c** but for SAM/linker–PARP contacts (699 Å^2^).

**Extended Data Fig. 4:**
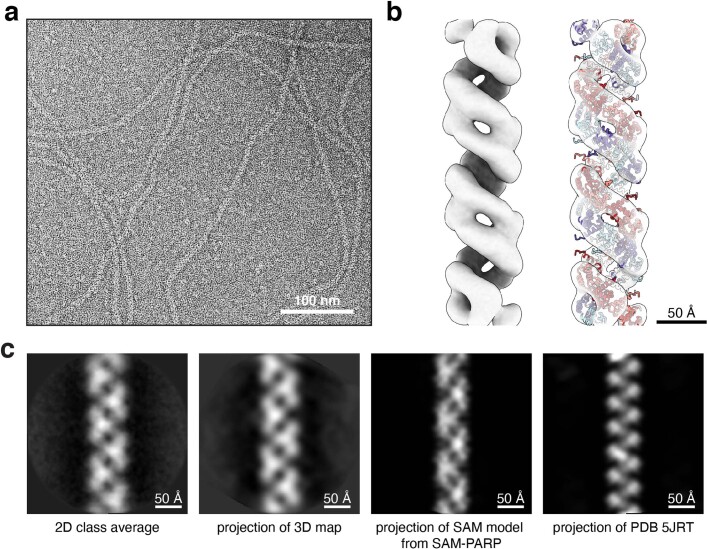
Negative-stain EM of TNKS2 SAM shows a two-start helix. **a**, Crop of a representative negative-stain electron micrograph (of 67) showing TNKS2 SAM filaments. **b**, 3D map of TNKS2 SAM alone (left) and rigid-body fitted with the SAM domain from the SAM-PARP model (right). **c**, Comparison between a 2D class average, a projection of the SAM domain filament 3D map from b, a projection of the SAM domain from the SAM-PARP model, and a projection of the single-stranded TNKS2 SAM DH902/924RE filament crystal structure (PDB ID 5JRT^4^); scale bars, 50 Å. The features of both the 2D class average and the projection of the 3D map obtained for TNKS2 SAM show clear similarity to those of the two-start SAM domain helix from the SAM-PARP model, as opposed to the single-start helix from the crystal structure. This demonstrates that the TNKS2 SAM domain autonomously forms two-start helices.

TNKS2 SAM–PARP forms a double-helix of antiparallel, left-handed protofilaments (Fig. 1b,c). This organization contrasts the single-stranded SAM domain helices observed in X-ray crystal structures for both tankyrase^4,5^ and other proteins^19,20^. Attachment of the two protofilaments gives rise to a minor and major helical groove (Fig. 1b,c). SAM–SAM head-to-tail contacts within each protofilament match those observed in previously determined crystal structures and involve the canonical end-helix and mid-loop surfaces (Fig. 2a,c). Interprotofilament contacts involve a distinct surface situated between the end-helix and mid-loop regions (Fig. 2a,d). As protofilaments are subtly offset against each other, each SAM domain contacts two SAM domains in the antiparallel protofilament (Fig. 1c and 2a,d). Negative-stain EM confirmed that double-helical polymerization is SAM-domain intrinsic and not dependent on the adjacent PARP domain (Extended Data Fig. 4). Therefore, single-start helices observed in crystallo^4,5^ are probably selected during crystallization.

**Fig. 3:**
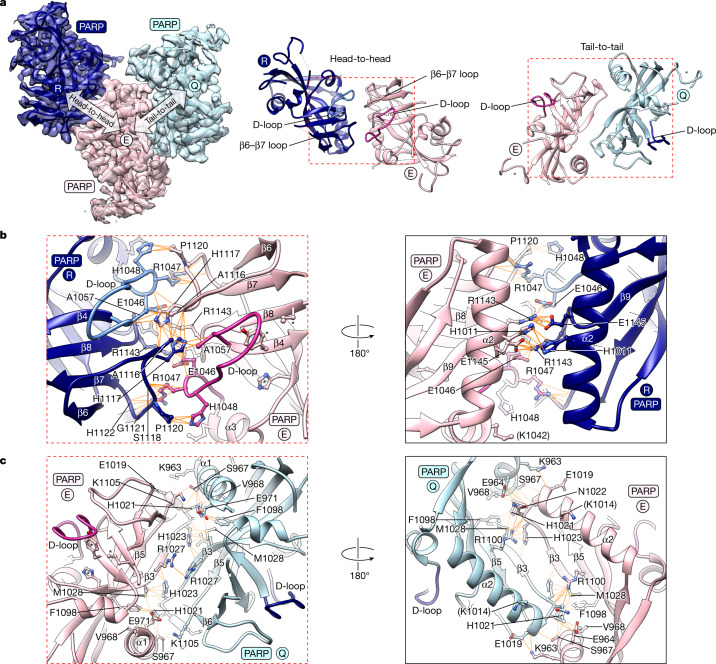
PARP–PARP domain contacts within the TNKS2 SAM–PARP filament. **a**, Cryo-EM map and model showing three adjacent PARP domains (left). Arrows indicate PARP–PARP head and tail interfaces. Overviews of the PARP–PARP interfaces in cartoon representation are also shown (right). **b**, Detailed cartoon representation of PARP–PARP head contacts (721 Å^2^), with key interacting residues shown as sticks and bonds indicated by orange lines. **c**, As for **b** but for PARP–PARP tail contacts (776 Å^2^). Residues of the catalytic H-Y-E triad are marked with asterisks, and letters indicate different protomers.

The PARP domains protrude outwards (Fig. 1b,c), connected to the SAM domains via an incompletely resolved, partially flexible linker (Fig. 1d), and decorate the major groove of the SAM double helix (Fig. 1b,c). PARP domains extending from one SAM protofilament do not contact each other but alternately intercalate with those extending from the antiparallel protofilament on the opposite side of the major groove (Fig. 1b,c). This architecture creates two distinct, alternating PARP–PARP head-to-head^18^ and tail-to-tail interfaces (Fig. 1b,c and Fig. 3a). The NAD^+^ co-substrate-binding (donor) and protein or PAR substrate-binding (acceptor) sites remain fully accessible on the filament periphery (Fig. 1b). The acceptor site is poorly resolved (Fig. 1d and Extended Data Fig. 2e), possibly because it needs to flexibly adapt to different protein substrates or the growing PAR chain. The central SAM and peripheral PARP domain assemblies are stably oriented relative to each other, owing to a recurring interface formed between the SAM/linker of one protomer and the PARP domain of an adjacent protomer within the same protofilament (Fig. 1b,c and 2e).

## SAM domain interprotofilament interface

The SAM interprotofilament surface is formed by helix α2 and the α4–α5 loop (Fig. 2d). Given D1 symmetry, these interactions are reciprocal. The main contact is conferred by R896^α2^ in two alternative conformations. In one conformation, the two R896^α2^ side chains stack against each other (Fig. 2d). In the other, R896^α2^ contacts D892^α2^, I893^α2^, the L890^α2^ side and main chains and the main chain of H889^α2^. A reciprocal contact between the N918^α4–α5loop^ side chains further contributes to the interface (Fig. 2d). The cryo-EM structure explains how R896^α2^ contributes to polymerization of the isolated TNKS2 SAM domain^4^.

## SAM/linker–PARP domain interface

In the SAM/linker–PARP domain interface, helix α1^SAM^ abuts β1^PARP^ and preceding residues (Fig. 2e). A single-turn helix in the SAM–PARP linker steeply redirects the polypeptide chain by approximately 120°. The linker binds to a surface on the ‘back’ of the PARP domain relative to its active site: G939^linker^ contacts W1006^PARPα2^, and L940^linker^ interacts with W1006^PARPα2^, Y1148^PARPβ9^ and E1150^PARPβ9^ via its side chain and K999^PARPβ2^ via its main chain (Fig. 2e). L944^linker^ binds to L958^PARPβ1^, E964^PARPα1^ and K999^PARPβ2^ (Fig. 2e).

## PARP–PARP domain interfaces

**Extended Data Fig. 5:**
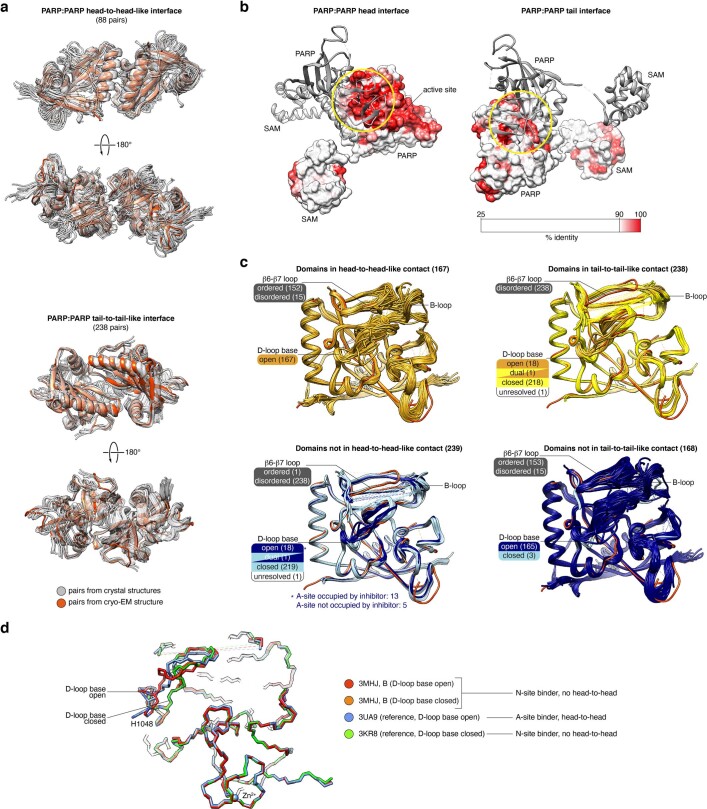
PARP:PARP contacts from crystal structures resembling head-to-head and tail-to-tail interactions. **a**, Superimposition of TNKS/TNKS2 PARP domain pairs in head- and tail-like interactions in crystal structures, as identified by PISA^21^. Domains are shown in transparent grey cartoon representation. PARP domains in head and tail interactions from the TNKS2 SAM-PARP filament are shown in orange red. **b**, Conservation of tankyrase PARP:PARP head and tail interfaces. The surface of a TNKS2 SAM-PARP protomer was coloured by percentage identity as indicated by the colour scale, using the alignment shown in Extended Data Fig. 6, omitting the non-tankyrase ARTD family members. SAM-PARP protomers interacting with the PARP domain of the reference protomer via the head or tail surfaces are shown in grey cartoon representation. The PARP:PARP head surface is as highly conserved as the enzyme active site. **c**, Superimposition of unique TNKS and TNKS2 PARP domains from the crystal structures that either engage (in tones of yellow) or do not engage (in tones of blue) in head- or tail-like contacts. A PARP domain from the TNKS2 SAM-PARP filament is shown in orange red. Domains are shown in worm representation. The D-loop base can adopt either an open (golden-yellow or dark blue) or closed (pale yellow or light blue) conformation. Of the domains not in a head-like contact and with an open D-loop base, most (13 out of 18) feature a small-molecule ligand in the A-site. The remaining 5 domains (with N-site binders) show electron densities in the A-site not attributable to the small-molecule inhibitor. Note that head- and tail-like PARP:PARP interactions do not co-occur in any crystal structure. **d**, Superimposition of TNKS2 PARP domains from the indicated crystal structures. Amino acid backbones and the H1048^TNKS2^ side chains are shown. One of the two chains in 3MHJ^31^ (no head-like contact, N-site binder) shows a D-loop base in two alternate conformations, open and closed, suggesting that in the absence of an A-site binder, the D-loop can sample open and closed conformations. See Supplementary Table 1 for PDB codes and analysis.

PARP domains engage in alternating PARP–PARP head-to-head and tail-to-tail contacts (Fig. 1b,c and Fig. 3). The former resembles a dimer observed in numerous TNKS/TNKS2 PARP domain crystal structures and recently proposed to regulate tankyrase catalytic activity^18^ (also see Extended Data Fig. 5a). With D1 symmetry, adjacent PARP domains are related by a 180° rotation and reciprocally engage the same surfaces (Fig. 3).

The head interface occurs close to the donor site, with main contact areas provided by helix α2, the donor loop (D-loop) atop the NAD^+^-binding site, the C-terminal portion of the β6–β7 loop and the β8–β9 loop (Fig. 3b). H1117^β6–β7loop^, R1143^β8–β9loop^, H1011^α2^ and A1116^β6–β7loop^ interact homotypically. Several residues at the ‘base’ of the D-loop contact the β6–β7 loop in the adjacent PARP domain: H1048^D-loop^ binds to P1120^β6–β7loop^; R1047^D-loop^ contacts the main chain of S1118^β6–β7loop^, P1120^β6–β7loop^, G1121^β6–β7loop^ and H1122^β6–β7loop^. E1046^D-loop^ binds to H1117^β6–β7loop^ and R1143^β8–β9loop^. A1057^D-loop^ interacts with A1116^β6–β7loop^, and the main chain of both residues is engaged by H1117^β6–β7loop^. R1143^β8–β9loop^ forms a salt bridge with E1145^β8–β9loop^. Distal to the active site, the H1011^α2^ side chains interact homotypically (Fig. 3b).

The tail interface involves reciprocal contacts formed by the α2–β3 loop, strands β3, β5, the β5–β6 loop and helices α1 and α2 (Fig. 3c). The α2–β3 loop is an interaction hotspot, contributing three consecutive contact residues: H1021^α2–β3loop^, N1022^α2–β3loop^ and H1023^α2–β3loop^. Of these, H1021^α2–β3loop^, the most buried interface residue, contacts E964^α1^, S967^α1^, V968^α1^, E971^α1^, F1098^β5^ and M1028^β3^. At the interface centre, two R1027^β3^ side chains interact homotypically (Fig. 3c).

## PARP domain conformational changes

**Fig. 4:**
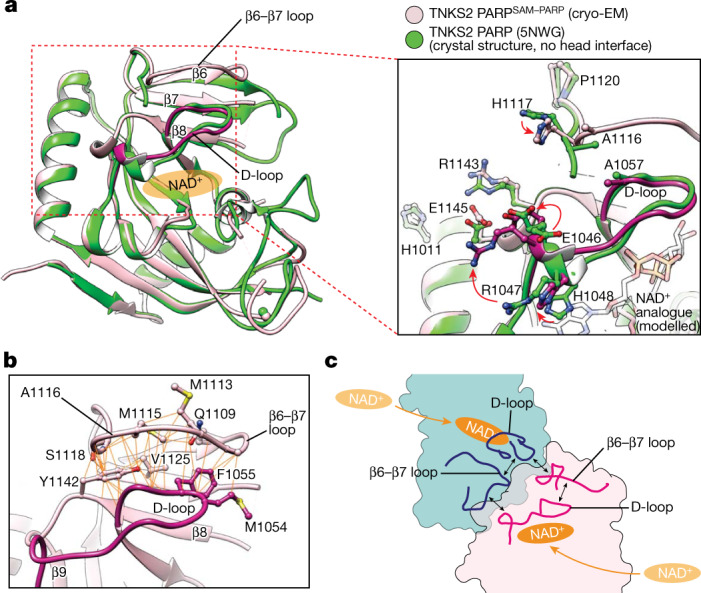
Conformational changes induced by PARP–PARP head interactions. **a**, PARP domain from the filament (pink, with D-loop in magenta) superimposed onto the crystal structure of TNKS2 PARP not in a head-like crystal contact (green, 5NWG^33^). Cartoon representation with the NAD^+^-binding site indicated (left), and a magnified view with selected head interface residues as sticks (right) are shown. The red arrows indicate conformational changes. The NAD^+^ analogue BAD was placed by superimposing the PARP1–BAD complex (6BHV^22^). **b**, Intramolecular D-loop–β6–β7 loop contacts. **c**, Schematic representation of intermolecular and intramolecular loop interactions at the PARP–PARP head interface. The double-headed arrows indicate interactions.

To explore the conformational effect of PARP–PARP interactions, we mined the Protein Data Bank (PDB) using the Protein Interfaces, Surfaces and Assemblies (PISA) tool^21^, identifying 88 PARP domain pairs in head-like contacts and 238 in tail-like contacts (Extended Data Fig. 5a and Supplementary Table 1). The two contacts did not co-occur in any crystal structure. Head-like contacts appeared to induce (1) an open conformation of the D-loop ‘base’ and (2) a fully ordered β6–β7 loop atop the D-loop (Fig. 4a, Extended Data Fig. 5c and Supplementary Table 1), as observed by cryo-EM. Although other determinants of D-loop conformation probably exist, such as small-molecule ligands (see Discussion), these observations suggest that the head contact opens the adenosine subsite of the NAD^+^ pocket.

**Extended Data Fig. 6:**
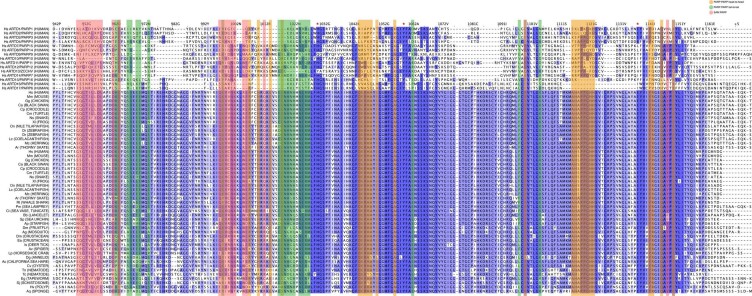
Multiple sequence alignment of catalytic domains from tankyrase homologues and ARTD family members. The indicated tankyrase orthologues and paralogues as well as Diphteria-toxin like ADP-ribosyltransferase (ARTD) family members were aligned and sequences coloured by percentage identity. Numbering above the alignment corresponds to human TNKS2. Sequences not aligning with the reference sequence (human TNKS2) were deleted for clarity. Buried residues in the PARP:PARP head-to-head, tail-to-tail and SAM:PARP interfaces, as defined by the PISA server^21^, are shaded in orange, green and red, respectively. See Methods for accession numbers.

The D-loop base opening is probably brought about by engagement of E1046^D-loop^, R1047^D-loop^ and H1048^D-loop^ in a head contact (Fig. 4a). H1048^D-loop^ swings out from a position in which it would otherwise clash with the adenine of the donor site NAD^+^ (Fig. 4a), immediately suggesting that formation of the head interface may displace H1048^D-loop^ from the donor site and instead enable it to stack with the adenine moiety of NAD^+^ (Extended Data Fig. 3b). A structured β6–β7 loop may be induced by repositioning of H1117^β6–β7loop^, which intermolecularly interacts with the D-loop of the adjacent head PARP domain (Fig. 4a). The β6–β7 loop also makes extensive intramolecular D-loop contacts (Fig. 4b). We hypothesize that reciprocal interactions between the β6–β7 loop and D-loop in the PARP–PARP head interface, as well as intramolecular contacts, induce an active conformation across two adjacent PARP domains (Figs. 3a and 4c). Of the two PARP–PARP interfaces, which appear unique to the tankyrases (Extended Data Fig. 6), the head interface is particularly highly conserved, to the same extent as the enzyme active site, suggesting a major regulatory role (Extended Data Figs. 5b and 6).

## Functions of PARP–PARP head contacts

We next probed the regulatory role of the domain interfaces by structure-guided mutagenesis. Although His_6_-MBP–TNKS2 SAM–PARP mutant variants displayed modest differences in thermal stability, as expected, melting was not observed at temperatures used in subsequent biochemical assays (Extended Data Fig. 9e). We expressed MYC_2_-tagged full-length TNKS2 variants in mammalian cells. We next assessed their activity using a WNT–β-catenin-responsive reporter assay and probed PARylation activity in anti-MYC immunoprecipitates^4^.

**Fig. 5:**
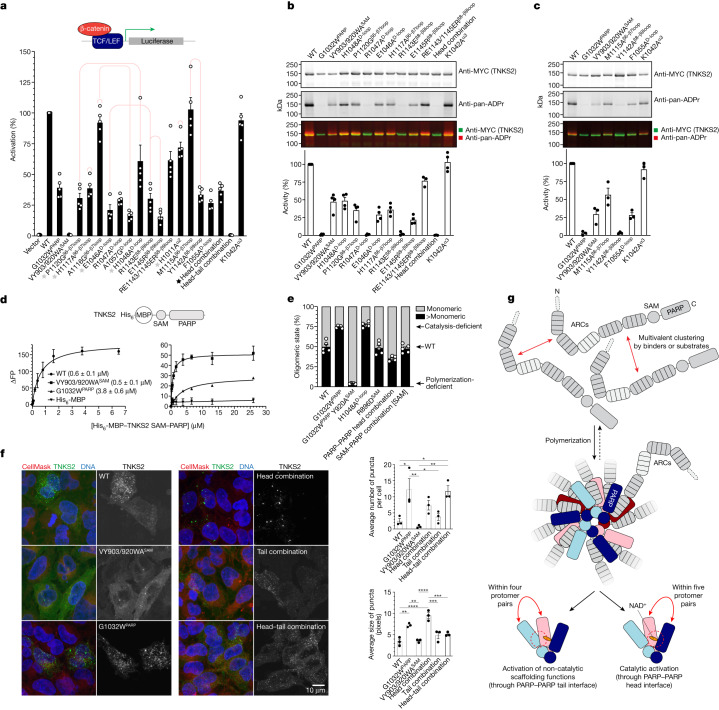
PARP–PARP domain interactions control tankyrase function. **a**, β-catenin-responsive luciferase reporter assay to analyse the roles of PARP–PARP head and D-loop interactions (*n* = 5 independent experiments (*n* = 4 for E1046A^D-loop^); individual data points and means; error bars indicate s.e.m.). The red lines denote side-chain interactions between mutated residues. The black star denotes the combination of mutations labelled by a grey star. K1042A^α3^ is a control mutation outside the head interface. See Extended Data Fig. 7 for reporter assays probing other interfaces and for expression levels. WT, wild type. **b**,**c**, Endogenous PARylation of PARP–PARP head interface (**b**) and loop contact mutants (**c**) analysed by western blotting of immunoprecipitated TNKS2 variants (*n* = 4 (**b**; *n* = 3 for RE1143/1145ER^β8–β9loop^ and head combination) or *n* = 3 (**c**) independent experiments; individual data points and means; error bars indicate s.e.m.). See Extended Data Fig. 8 for in vitro PARylation and PARP activity assays probing other interfaces. ADPr, ADP-ribose. **d**, Fluorescence polarization (FP) to analyse binding of His_6_-MBP–TNKS2 SAM–PARP variants to BAD (*n* = 3 independent experiments; error bars indicate s.e.m.; *K*_d_ with s.e. is also indicated). See Extended Data Fig. 9g for protein SDS–PAGE. **e**, Mass photometry to analyse oligomerization of His_6_-MBP–TNKS2 SAM–PARP variants. The percentages of monomeric or larger than monomeric particles are shown (*n* = 5 independent experiments; individual data points and means; error bars indicate s.e.m.). See Extended Data Fig. 9a,b for probability density graphs and additional mutant variants. **f**, Representative fluorescence micrographs of mCitrine–TNKS2-expressing HeLa cells (left), and quantification of micrographs (right) (*n* = 3 independent experiments; individual data points and means; error bars indicate s.e.m.; statistical significance as per one-way ANOVA with Tukey’s multiple comparisons test; ****P < 0.0001; ***P ≤ 0.001; **P ≤ 0.01; *P ≤ 0.05; no label, *P *> 0.05). See Extended Data Fig. 9c,d for data from the +tankyrase inhibitor condition. **g**, Model for the activation of catalytic and non-catalytic tankyrase functions by polymerization. The dashed arrow indicates de-polymerization. Double-headed red arrows indicate interactions.

**Extended Data Fig. 7:**
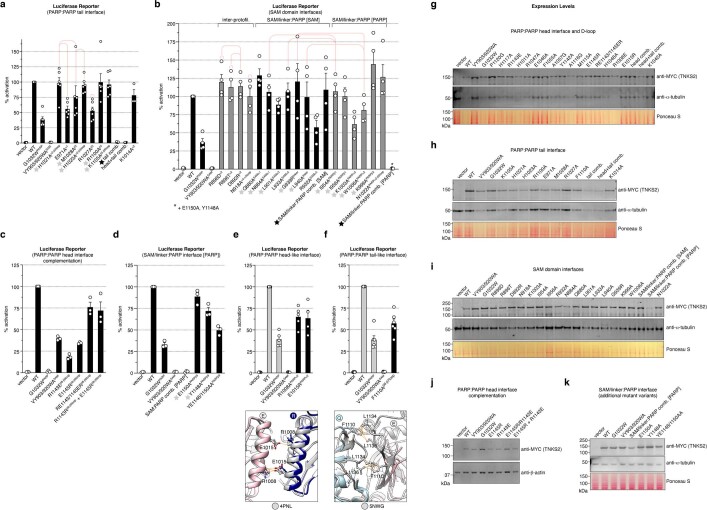
Luciferase reporter assays to analyse contributions of domain interfaces to tankyrase function. **a-d**, β-catenin-responsive TOPFlash luciferase reporter assay to analyse the contributions of PARP:PARP tail and head, inter-protofilament, and SAM/linker:PARP interactions (a, n = 6 (3 for K1014A); b, n = 4; c, n = 3; d, n = 3 independent experiments; individual data points and means; error bars, SEM). Red lines denote side chain interactions amongst mutated residues. The black star denotes the combination of those mutations labelled by a grey star for each set. K1014A^α2^ is a control mutation outside the tail interface. N1022A^PARPα2-β3loop^ was designed to disrupt a potential contact with the SAM domain of the protomer interacting in the PARP:PARP head mode, but also contributes to the PARP:PARP tail interface. The ≈45% reporter reduction conferred by the R932A^SAMα5^ mutation is likely due to its moderate impact on polymerisation^4^. Given that the combined SAM domain mutations disrupting the SAM/linker:PARP interface did not affect reporter activation, the K1003A^PARPα2^ mutation likely acts through a mechanism that is independent of the SAM/linker:PARP interaction. **e**, Analysis of the contributions of selected head-like PARP:PARP domain contacts observed in crystal structures. Top, β-catenin-responsive TOPFlash luciferase reporter assay (n = 5 independent experiments (4 for E1015R); individual data points and means; error bars, SEM). The data for the vector, WT, G1032W^PARP^ and VY903/920WA^SAM^ controls are the same as those shown in Fig. 5a as mutants were analysed in the same set. Bottom, superimposition of head-to-head pair from the cryo-EM structure (cartoon in colour) and the head-to-head-like pair from crystal structure 4PNL^67^ (cartoon in grey, chains shown on the left superimposed). Selected amino acids are shown in stick representation with contacts drawn as orange lines. **f**, as e, but for tail-tail and tail-to-tail-like interface. Luciferase reporter controls are the same as shown in a as mutants were analysed in the same set (n = 6 independent experiments; individual data points and means; error bars, SEM). The reference crystal structure is 5NWG^33^. Note that F1110^β6-β7loop^ sits in the β6-β7 loop. **g–k**, Samples from a third and fourth technical replicate in the luciferase reporter assays were probed by Western blotting for MYC-TNKS2 and α-tubulin or β-actin to assess the expression levels of the TNKS2 mutant variants. Ponceau S staining of the membrane serves as additional loading control. Data from one representative experiment (of at least three with similar results) are shown.

Overexpression of TNKS2 robustly activated the reporter. Reporter activation was reduced by approximately 60% upon full catalytic inactivation (G1032W^PARP^) and completely abolished by loss of polymerization (VY903/920WA^SAM^)^4^, which in turn reduced catalytic activity by approximately 50% (Fig. 5a,b). This reflects a combination of both PARylation-dependent and PARylation-independent (scaffolding) functions of TNKS2 (ref. ^4^). The P1120G^β6–β7loop^ and H1117A^β6–β7loop^ mutations within the β6–β7 loop, designed to disrupt intermolecular interactions with the D-loop, reduced reporter activation to an extent comparable to that of G1032W^PARP^ and auto-PARylation by more than 60% (Fig. 5a,b). Mutation of corresponding contact residues in the D-loop, E1046A^D-loop^ and R1047A^D-loop^, also strongly reduced reporter activation and auto-PARylation (Fig. 5a,b). The A1057G^D-loop^ mutation was similarly disruptive in the reporter, but probably by rendering the D-loop more flexible, as mutation of its interacting residue A1116^β6–β7loop^ showed little effect (Fig. 5a). Mutating H1048^D-loop^ had a weaker effect than mutating its interacting P1120^β6–β7loop^, potentially reflecting its proposed complex role in either blocking or supporting NAD^+^ binding, depending on the base conformation of the D-loop, and contributing to the PARP–PARP head interface (Figs. 3b and 5a,b and Extended Data Fig. 3b). Charge reversal of the salt-bridge partners (R1143E^β8–β9loop^ and E1145R^β8–β9loop^) strongly reduced reporter activity and auto-PARylation (Fig. 5a,b). Combination of these two mutations within the same protein or co-expression of the two mutant variants, expected to re-establish the salt bridge, partially restored function (Fig. 5a,b and Extended Data Fig. 7c). An H1011A^α2^ mutation to disrupt a homotypic interaction distal to the active site moderately reduced reporter activation (Fig. 5a). Combination of five head interface mutations (‘head combination’: H1011A^α2^, E1046A^D-loop^, H1117A^β6–β7loop^, P1120G^β6–β7loop^ and R1143A^β8–β9loop^), which rendered tankyrase catalytically inactive, did not further reduce reporter activation below PARP-inactive (G1032W^PARP^) levels (Fig. 5a,b), in line with a joint role of these residues in catalysis.

**Extended Data Fig. 8:**
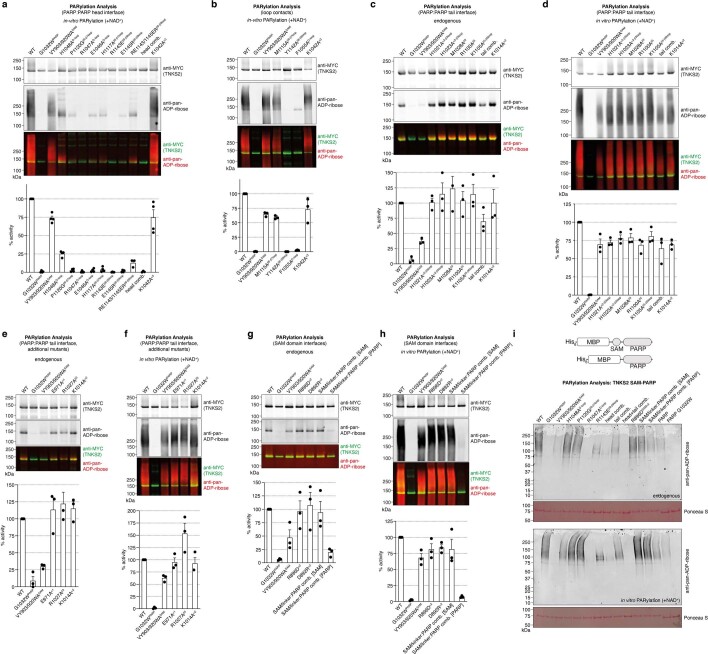
Analysis of contributions of domain interfaces to tankyrase catalytic activity. **a-h**, Assessment of PARylation status by Western blotting of immunoprecipitated TNKS2 interface mutant variants, either directly after immunoprecipitation (endogenous) or upon *in vitro* PARylation by incubation with NAD^+^. The PARylation signals from 3 [b-h] or 4 [a, except for RE1143/1145ER^β8-β9loop^ and head comb., where n = 3) independent experiments were quantified (individual data points and means; error bars, SEM). **i**, Assessment of PARylation status by Western blotting of purified His_6_-MBP-TNKS2 SAM-PARP proteins produced in *E. coli*, either directly after purification (endogenous, top) or upon *in vitro* PARylation by incubation with NAD^+^ (bottom). ADP-ribose was detected using an anti-pan-ADP-ribose reagent; protein loading was assessed by Ponceau S staining of the membrane. One representative out of three independent experiments with similar results is shown.

We further assessed the relevance of intramolecular β6–β7 loop–D-loop interactions (Fig. 4b,c). Two mutations to render the β6–β7 loop more flexible, M1115A^β6–β7loop^ and A1116G^β6–β7loop^ showed little to no effect on the reporter (Fig. 5a), but M1115A^β6–β7loop^ reduced ADP-ribosylation by approximately 50% (Fig. 5c and Extended Data Fig. 8b). Both mutation of Y1142^β8–β9loop^ or F1055^D-loop^, which indirectly or directly mediate D-loop–β6–β7 loop interactions, respectively, strongly reduced reporter and catalytic activities (Figs. 4b and 5a,c and Extended Data Fig. 8b).

Upon in vitro PARylation, the impact of mutations for which we observed intermediate catalytic reduction was exacerbated, further confirming a role of the PARP–PARP head interface in catalysis (Extended Data Fig. 8a,b).

To directly test whether tankyrase polymerization facilitates NAD^+^ binding, we measured the affinity of His_6_-MBP–TNKS2 SAM–PARP for a fluorescently labelled, non-hydrolysable NAD^+^ analogue (benzamide adenine dinucleotide (BAD))^22^ by fluorescence polarization. The wild-type variant bound BAD with an apparent affinity of 0.6 ± 0.1 μM (Fig. 5d). Loss of polymerization (VY903/920WA^SAM^) reduced the Δfluorescence polarization magnitude, due to the expected decrease in size, but did not change the dissociation constant (*K*_d_; 0.5 ± 0.1 μM). The inactivating G1032W^PARP^ mutation reduced BAD binding to 3.8 ± 0.6 μM (Fig. 5d). Provided that filaments formed by the wild-type variant were of sufficient length to enable PARP–PARP head interactions, increased affinity for NAD^+^ is unlikely to be responsible for the polymerization-dependent catalytic switch.

## Functions of PARP–PARP tail contacts

We next assessed the role of the PARP–PARP tail interface. Except for the E971A^α1^ and R1027A^β3^ mutations, individual tail interface mutations did not reduce reporter activation or catalysis (Extended Data Fig. 7a), nor did individual mutations impair catalysis (Extended Data Fig. 8c–f). However, combination of the H1021A^α2–β3loop^, H1023A^α2–β3loop^, M1028A^β3^, R1100A^β5^ and K1105A^β5–β6loop^ mutations (‘tail combination’) fully abolished reporter activation, just as disruption of polymerization (Extended Data Fig. 7a), whereas the effect on catalysis remained relatively modest (Extended Data Fig. 8c,d). These findings suggest that PARP–PARP tail interactions collectively contribute to an unknown, primarily non-catalytic (scaffolding) mechanism downstream of polymerization.

## Probing novel SAM and linker contacts

Disruption of the SAM–SAM interprotofilament interface had no measurable effect on reporter activation by TNKS2 and catalysis (Extended Data Figs. 7b and 8g,h), in line with our previous observations^4^. Individual mutations on either face of the SAM/linker–PARP interface only moderately affected reporter activation (Extended Data Fig. 7b; see figure legend for details). Combination of five SAM domain mutations within the SAM/linker–PARP interface (Q880A^SAMα1^, N884A^SAMα1^, L901A^SAMα3^, G939R^linker^ and L940A^linker^) had no measurable effect on reporter activation (Extended Data Fig. 7b) and PARP activity (Extended Data Fig. 8g,h).

However, combining seven PARP domain mutations within the SAM/linker–PARP interface (I954A^PARPβ1^, I956A^PARPβ1^, K1003^PARPα2^, W1006A^PARPα2^, K999A^PARPβ2^, E1150A and Y1148A) completely abolished TNKS2-dependent reporter activation (Extended Data Fig. 7b). Given that extensive mutation of the interface on the SAM domain side was tolerated, mutations of the PARP domain must impinge on another function distinct from the SAM/linker–PARP interaction. This function is potentially identical to that of the contiguous PARP–PARP tail surface (Fig. 2b). Contrary to the tail surface, however, combined PARP domain mutations within the SAM/linker–PARP interface nearly completely abolished catalytic activity (Extended Data Fig. 8g,h).

## PARP–PARP contacts stabilize polymers

**Extended Data Fig. 9:**
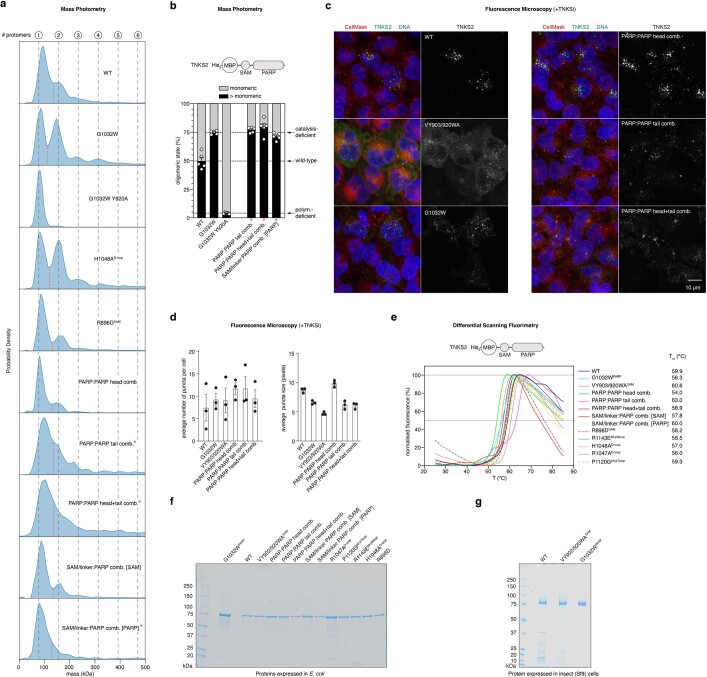
Analysis of TNKS2 oligomerisation, localisation and structural integrity. **a**, Mass photometry analysis of the indicated His_6_-MBP-TNKS2 SAM-PARP variants. Representative probability density graphs from one out of five experiments are shown for each variant, with theoretical molecular weights of species containing one to six subunits indicated by dotted lines. Dashed vertical red lines indicate boundaries between monomeric and >monomeric species used for quantification. The three species marked with asterisks have poorer separation between peaks due to a higher tendency of molecules to repeatedly bind and unbind to and from the glass surface (see Methods for details). **b**, Analysis of mass photometry data for additional PARP domain mutant variants as in Fig. 5e (n = 5 independent experiments; individual data points and means; error bars, SEM). Data for controls (WT, G1032W^PARP^, G1032W^PARP^ Y920A^SAM^) are identical to Fig. 5e and shown again for reference purposes. As for a, the three species marked with asterisks should be interpreted with caution. **c**, Fluorescence microscopy as in Fig. 5f, but after application of a tankyrase catalytic inhibitor. **d**, Quantification of c (n = 3 independent experiments; individual data points and means; error bars, SEM). **e**, Differential scanning fluorimetry of the indicated variants of His_6_-MBP-TNKS2 SAM-PARP. Data are means from three parallel technical replicates. **f**, **g**, Coomassie-stained SDS-PAGE gels of purified His_6_-MBP-TNKS2 SAM-PARP fusion proteins for quality control. Sufficiently high protein yield did not require repeated purifications for most of the variants (n = 1).

We used mass photometry to explore whether PARP domain contacts contribute to polymerization, measuring the size of His_6_-MBP–TNKS2 SAM–PARP at the single-molecule level. Upon dilution to 50 nM (necessary for the method), monomers constituted the predominant species (approximately 50%), followed by dimers and trimers (Fig. 5e and Extended Data Fig. 9a). Catalytic inactivation (G1032W^PARP^) substantially increased the dimer population and gave rise to well-defined trimer, tetramer and detectable pentamer peaks (approximately 75% multimeric (that is, >monomeric); Fig. 5e and Extended Data Fig. 9a), in line with polymerization inhibition by auto-PARylation^3^. Additionally breaking SAM–SAM head-to-tail contacts (G1032W^PARP^ Y920A^SAM^)^4^ resulted in nearly 100% monomers (Fig. 5e and Extended Data Fig. 9a). Although the PARP–PARP head combination mutant was just as catalytically impaired as G1032W^PARP^ (see above), it appeared less polymeric (approximately 35% >monomeric), also than the wild-type protein, indicating that PARP–PARP head interactions contribute to polymerization. Note, however, that the studied species are probably dissociation products of larger polymers lost by dilution as PARP–PARP contacts are not expected to form in these very short filaments (see Discussion). The H1048A^D-loop^ mutation conferred increased polymerization, comparable with G1032W^PARP^, suggesting that the associated reduction in catalytic activity is sufficient to favour polymerization (Fig. 5e and Extended Data Fig. 9a). Mutation of the interprotofilament contact (R896D^SAM^) and SAM/linker–PARP interface (on the SAM domain side) did not detectably reduce polymerization (Fig. 5e and Extended Data Fig. 9a). Variants containing combined PARP domain mutations in the PARP–PARP tail and SAM/linker–PARP interfaces appeared less monomeric, but presented with a higher tendency to stick to the glass surface, potentially due to aggregation, hindering reliable quantification (Extended Data Fig. 9a,b and Supplementary Video 1).

## PARP–PARP contacts control localization

We generated HeLa cells stably and inducibly expressing mCitrine-tagged TNKS2 variants and used confocal microscopy to explore whether domain interactions contribute to polymerization-dependent punctate tankyrase localization^4,5^. In line with previous findings^4^, TNKS2 formed cytoplasmic puncta that increased in number and size by catalytic inactivation and were rendered diffuse by polymer-breaking mutations (Fig. 5f). Whereas the PARP–PARP tail interface combination mutant displayed a similar punctate localization as wild-type TNKS2, combined PARP–PARP head interface mutations paradoxically increased the number and particularly the size of puncta despite reducing polymerization in mass photometry (Fig. 5e,f). Puncta may therefore not necessarily be direct correlates of polymerization and instead also reflect polymerization effector functions and the action of cellular factors, such as components of the WNT–β-catenin signalling machinery^10,23^. The increased puncta size observed for the PARP–PARP head interface mutant was suppressed by mutation of the tail interface (Fig. 5f). Pharmacological inhibition of tankyrase promoted overall puncta formation^23^ (Extended Data Fig. 9c,d).

## Discussion

**Extended Data Fig. 10:**
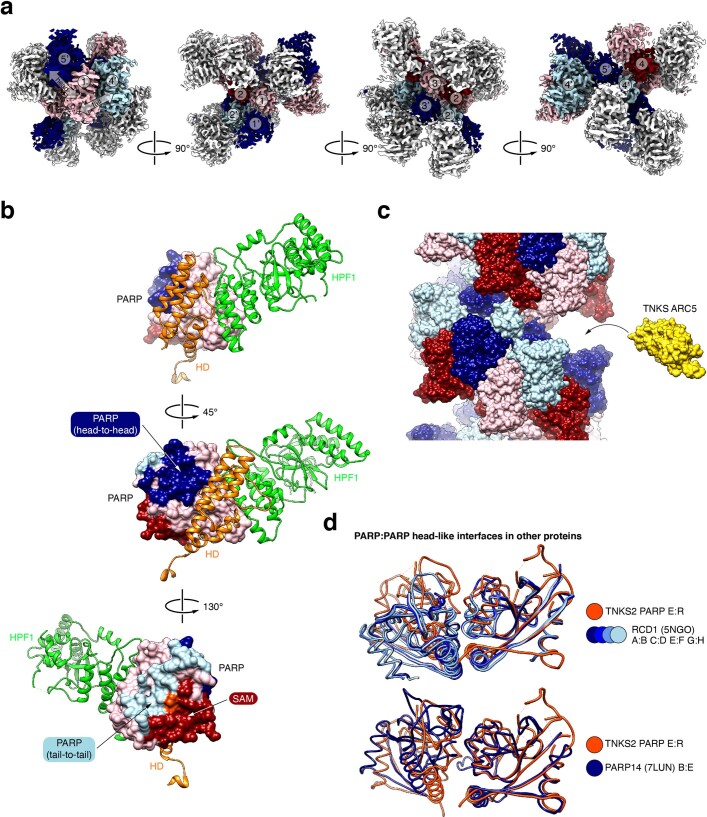
Regulatory assembly and surfaces on TNKS2 SAM-PARP. **a**, Cryo-EM map of five consecutive pairs of TNKS2 SAM-PARP protomers, numbered n (for sense strand) and n’ (for antisense strand). Three PARP domains are shown in colour to illustrate the PARP:PARP contacts. Four or five pairs of protomers, respectively, need to assemble for a PARP:PARP tail (1:4’) or head (1:5’) contact to be established. **b**, The regulatory surfaces corresponding to those engaged by the helical domain (HD) in PARP1-3/ARTD1-3 and HPF1 in PARP1&2/ARTD1&2 remain available in the TNKS2 SAM-PARP filament. The PARP domain from TNKS2 SAM-PARP is shown in surface representation, coloured as in Fig. 2b. The HD of human PARP2, shown in orange cartoon representation, and human HPF1, shown in green cartoon representation, were oriented by superimposing the PARP2 catalytic domains from the PARP2 HD-PARP-ABT888 complex structure (3KJD chain A^68^) and the PARP2 PARP-HPF1 complex structure (6TX3^30^). **c**, ARC5 could likely be accommodated in the available minor groove of TNKS2 SAM-PARP. The TNKS2 SAM-PARP filament model is shown in surface representation and coloured as in Fig. 1. TNKS ARC (from 5GP7^69^) is shown in yellow surface representation. **d**, PARP:PARP head-like interactions in crystal structures of other ADP-ribosyltransferases, identified by PISA^21^. Top, *A. thaliana* RCD1 (5NGO^70^). Bottom, human PARP14/ARTD8 (7LUN^71^). Domains are shown in worm representation with chain identifiers. The TNKS2 PARP:PARP head contact is shown in orange red.

SAM-domain-dependent tankyrase polymerization induces supramolecular assemblies to facilitate the recruitment of client proteins through avidity and promote catalytic PARP activity^3–5,18^. Here we demonstrate that polymerization gives rise to both PARP–PARP head and tail interfaces that collectively promote tankyrase catalysis, polymerization, contribute to both catalytic and non-catalytic functions of tankyrase in WNT–β-catenin signalling and determine subcellular localization (Fig. 5g). Our findings support a model in which the highly conserved, polymerization-induced PARP–PARP head interface triggers a conformational change to increase PARylation activity. Opening of the D-loop base agrees with a model proposed by Fan et al.^18^ based on PARP domain dimers observed in crystal structures, although the precise side-chain contacts differ in our cryo-EM structure^18^. Catalytic activation is probably brought about by the displacement of a negative regulatory histidine (H1048^TNKS2^ and H1201^TNKS^) from the adenosine subsite of the NAD^+^-binding pocket. We hypothesize that the active sites of adjacent PARP domains communicate through reciprocal intermolecular interactions across the PARP–PARP head interface (Fig. 4c), suggesting cooperativity. Given the high degree of conservation between TNKS and TNKS2, and their heteropolymerization^4^, this mechanism is probably shared by both paralogues, in agreement with experimental data^18^. Our PISA analysis also identifies head-like PARP–PARP domain interactions in crystal structures of other ADP-ribosyltransferases, namely, human PARP14 and *Arabidopsis thaliana* RCD1, suggesting that the activation mechanism may be more widely used (Extended Data Fig. 10d).

Although catalysis-inactivating mutations in the PARP domain or even full PARP domain deletion preserve a degree of reporter activation (indicative of non-catalytic functions)^4^, we paradoxically identified PARP domain point mutations that completely abolish tankyrase function in WNT–β-catenin signalling. We speculate that a PARP-domain-binding cellular factor suppresses tankyrase function by binding to a surface that is occluded when the PARP–PARP tail and SAM/linker–PARP interfaces are engaged.

Antiparallel double helices, potentially a general feature of SAM polymers^17,24^, may facilitate polymer nucleation, conferring sensitivity to concentration-dependent, non-polar self-assembly. As interacting PARP domains extend from opposite sides of the major groove, regulatory PARP–PARP contacts will only occur when protofilaments reach a length of at least four protomers (for tail interactions) or five protomers (for head interactions) (Extended Data Fig. 10a). This is less than the number of subunits per turn (approximately 7) given the ‘reach’ of interacting PARP domains from antiparallel strands towards each other, facilitated by SAM/linker–PARP interactions (Fig. 1c and Extended Data Fig. 10a). This minimal polymer length would impose a polymerization threshold to be surpassed for activation, thereby conferring robustness to tankyrase activation, limiting the noise in effector output by the transient formation of very short polymers. Long filaments may not be required for tankyrase activation, in line with the punctate localization of tankyrase. However, locally abundant multivalent substrates may hyper-induce tankyrase polymerization and tune catalysis towards the demand for PARylation (Fig. 5g). In the context of the filament, substrates bound to ankyrin repeat clusters of one tankyrase protomer may be PARylated by catalytic domains of other protomers. Other signalling systems use filamentous polymerization, such as the WNT–β-catenin signalling components Axin (AXIN1/2) and Dishevelled (DVL2)^25^, the IRE1 kinase in the unfolded protein response^26^, inflammasomes^27^ or metabolic enzymes^28^. This points towards shared molecular principles in the regulation of cellular processes by polymerization.

The SAM domain of tankyrase is sometimes compared with the helical domain that precedes the PARP1/2/3 catalytic domain^9^. Whereas the helical domain negatively regulates PARP activity by blocking NAD^+^ binding^22,29^, polymerization of the SAM domain positively regulates catalysis. The helical domain occupies an entirely distinct but adjacent surface to that engaged by the SAM or PARP domains in polymeric tankyrase (Extended Data Fig. 10b). Similarly, the surface equivalent to the binding site of the PARP1/2 regulator histone PARylation factor 1 (HPF1)^30^ also remains available in the tankyrase filament (Extended Data Fig. 10b) for potential interactions with other factors. An ankyrin repeat cluster emerging from the SAM domain could be accommodated across the minor groove of the SAM domain polymer and potentially control tankyrase PARP activity (Extended Data Fig. 10c).

The crystallographic capture of the D-loop in alternate conformations in the absence of a head-like PARP–PARP contact^31^ suggests that the D-loop can sample both conformations dynamically (Extended Data Fig. 5d). Binding of small-molecule inhibitors in the adenosine subsite appears to induce an open D-loop base conformation (Extended Data Fig. 5c and Supplementary Table 1). Akin to ‘reverse allostery’ observed for PARP1 (refs. ^9,22^), we hypothesize that binders to the adenosine site may promote polymerization by opening the D-loop base^32^, thereby facilitating the PARP–PARP head contact. It is also conceivable that bulkier inhibitors could be developed that extend into the PARP–PARP head interface.

## Methods

### Plasmids and cell lines used in this study

See Supplementary Table 2 for plasmids used in this study. Mutant variants of TNKS2 were either obtained by PCR-based site-directed mutagenesis on pLP-dMYC SD-*Hs* TNKS2 (NM_025235)^34^ or, for a subset of combination mutants, by gene synthesis (GenScript). See Supplementary Table 3 for nomenclature of combination mutant variants. The pcDNA5-FRT/TO-mCitrine vector was generated by first PCR-amplifying an mCitrine-coding fragment from pLP-mCitrine C1 SD (V3534)^35^. The template-derived fragment included a 5′ Kozak sequence and a 3′ linker (coding for GSGRA). PCR introduced a 5′ *HindIII* restriction site and 3′ *AscI*, *PacI* and *BamHI* restriction sites. The PCR product was inserted into pcDNA5-FRT/TO using the *HindIII* and *BamHI* sites. The *TNKS2* cDNA, excised from pLP-dMYC SD-*Hs* TNKS2 constructs^4,34^, was cloned into the vector using the *AscI* and *PacI* restriction sites.

Sf9 (*Spodoptera frugiperda*) insect cells were obtained from Thermo Fisher Scientific.

HEK293T cells were obtained from C. Lord (ICR, London). HeLa Flp-In T-Rex cells were a gift from S. Taylor (University of Manchester)^36^. Cell lines were authenticated at source and confirmed to be free of Mycoplasma contamination. HEK293T and HeLa Flp-In T-Rex cells (and derivatives) were maintained in a humidified incubator at 37 °C with 5% CO_2_ in DMEM supplemented with antibiotics (streptomycin sulfate and benzylpenicillin), 2 mM glutamine and 10% FBS (F7524, Sigma). To generate HeLa Flp-In T-Rex cell lines inducibly expressing mCitrine–TNKS2, parental cells were cultured under pre-selection by zeocin (50 μg ml^−1^) and blasticidin (4 μg ml^−1^). Parental HeLa Flp-In T-Rex were seeded at 8 × 10^4^ cells per well in six-well plates. Twenty-four hours later, cells were co-transfected with pOG44 (encoding Flp recombinase) and pcDNA5-FRT/TO-mCitrine–TNKS2, at a ratio of 9:1 (total of 2 μg), using Lipofectamine 2000 (Thermo Fisher Scientific), with a DNA:lipofectamine ratio of 1:2. Twenty-four hours post-transfection, cells were re-plated on a 10-cm dish and selected with hygromycin (200 μg ml^−1^) and blasticidin (4 μg ml^−1^), to allow resistant colonies to form. Colonies were then pooled and expanded to create a polyclonal cell line with stably integrated mCitrine–TNKS2 under a tetracycline-inducible promoter.

### Expression and purification of TNKS2 SAM–PARP^G1032W^ (867–1162) for EM

TNKS2 SAM–PARP^G1032W^ (867–1162) was produced recombinantly as a His_6_-MBP-Asn_10_-TEV fusion protein in *Escherichia coli* BL21-Codon Plus (DE3)-RIL (Stratagene) grown in Terrific Broth media. Expression was induced at an optical density at 600 nm (OD_600_) of 2.0 with 0.5 mM IPTG overnight at 18 °C. Cells were collected by centrifugation and resuspended in a buffer containing 50 mM Tris-HCl (pH 7.5), 0.5 M NaCl, 5 mM imidazole (pH 7.5; to enable nickel affinity purification with reduced background from the same lysate should MBP affinity purification give insufficient yields), 10 mM β-mercaptoethanol and protease inhibitors (1 mM PMSF, 1 μg ml^−1^ leupeptin, 1 μg ml^−1^ aprotinin and 1 μg ml^−1^ pepstatin A; 50 ml lysis buffer was added to a pellet from each 1 l of culture). The cells were lysed by sonication using a Vibra-Cell sonicator (Sonics & Materials) and centrifuged to remove insoluble cellular debris. The lysate was filtered through a 0.45-μm filter and loaded onto a 5-ml MBPTrap HP affinity column (GE Healthcare/Cytiva). The column was washed with at least 5 column volumes (CV) of wash buffer (as lysis buffer, but without protease inhibitors). The His_6_-MBP-Asn_10_ fusion protein was eluted with a linear α-methyl glucoside (AMG) gradient (0–2 M) in a buffer also containing 50 mM Tris-HCl (pH 7.5), 0.5 M NaCl and 10 mM β-mercaptoethanol. The protein was incubated with recombinant MBP-tagged TEV protease overnight to cleave the His_6_-MBP-Asn_10_ tag, and dialysed against 50 mM Tris-HCl (pH 7.5), 0.5 M NaCl and 10 mM β-mercaptoethanol. As removal of the cleaved tag by a subtractive affinity purification reduced filament yield, the cleaved tag was not removed from the sample prior to imaging. Aliquots of TNKS2 SAM–PARP^G1032W^ were flash-frozen in liquid nitrogen and stored at −80 °C.

### Cryo-EM grid preparation and data collection

To prepare grids for cryo-EM, 3 μl of purified protein at approximately 25 μM, in a buffer containing 50 mM Tris-HCl (pH 7.5), 0.5 M NaCl and 10 mM β-mercaptoethanol, were applied onto previously glow-discharged Quantifoil Cu R 1.2/1.3 400 mesh carbon-coated grids for 30 s in a humidity-controlled Vitrobot Mark IV automatic plunge freezer (Thermo Fisher Scientific). Humidity was set to 100%, but regulation was turned off 10 min before grid preparation, and temperature was set to 18 °C. After a 30-s incubation, 2 μl of water were pipetted onto the protein solution and removed, and the process was repeated ten times to gradually lower the salt concentration. The grids were blotted using the Vitrobot Mark IV (Thermo Fisher Scientific) and plunged into liquid ethane cooled by liquid nitrogen. Vitrified grids were imaged in separate data collection sessions using Titan KRIOS transmission electron microscopes (Thermo Fisher Scientific) operated at 300 keV at the Electron Bio-Imaging Centre, Diamond Light Source, UK. A total of five datasets were collected with direct-detector cameras, four with a Gatan K2 Summit and one with a Gatan K3 camera in super-resolution counting mode using EPU software (Thermo Fisher Scientific, v1.14.0.54). Cryo-EM micrographs were collected as movies with a nominal pixel size of 1.06 Å (super-resolution dataset with a pixel size of 0.53 Å), a total accumulated dose of 40 electrons per Å^2^ and applied defocus values from −1.2 to −3.5 μm.

### Image processing

Movies were processed using RELION (v2.10 and 3.08)^37,38^. Images were motion-corrected and dose-weighted using MotionCor2 (ref. ^39^). Movies from the K3 super-resolution counting dataset were 2× binned at this stage, resulting in a pixel size of 1.06 Å. The contrast transfer function (CTF) was estimated using CTFFIND4 (ref. ^40^). Straight portions of filaments were manually selected as start–end coordinates and extracted with a box size of 400 pixels and an inter-box distance of 27.2 Å (asymmetric unit 2). Reference-free 2D classification was performed, and segments from suboptimal 2D class averages were discarded. Selected particles from good 2D classes from each dataset were combined (139,880 particles in total). To estimate the helical symmetry parameters, the best 2D class averages were padded in a larger box of 1,200 × 1,200 pixels for finer Fourier sampling, and their power spectra generated in Bshow (from Bsoft v1.9.5)^41,42^. The individual power spectra were then iteratively rotationally aligned using e2align2d.py (from EMAN2 v2.31)^43^, using the sum of three already vertically well-aligned power spectra as initial reference, and the sum of aligned spectra as reference for further alignment iterations. The final sum of power spectra was inspected using HELIXPLORER (http://rico.ibs.fr/helixplorer/; version March 2018) for determination of initial symmetry parameters (Extended Data Fig. 2b). The power spectra showed regularly spaced layer lines, indicating a pseudo-repeat of 94 Å. The first layer line from the equator showed a maximum near the meridian and indicated a pitch of approximately 94 Å, which was confirmed by inspecting the real-space 2D class average. A layer line with a peak on the meridian indicated an axial rise of approximately 13.5 Å, therefore enabling the number of units per turn to be calculated as approximately 7 (94/13.5). This symmetry was tested on a well-defined and verticalized 2D class average using Segclassreconstruct from SPRING (v0.86.1661)^44^.

Helical reconstructions were refined using 3D auto-refinement in RELION (v2.10 and 3.08)^45^. The first 3D auto-refinement step used a cylinder with an outer diameter of 152 Å as the initial reference. The helical parameters converged to a helical twist of −52.3° and a helical rise of 13.6 Å. The resulting 3D reconstruction suggested the presence of twofold symmetry, and applying D1 point group symmetry during 3D auto-refinement increased the resolution of the map. The subsequent refinement steps used the previously generated 3D reconstruction, low-pass filtered to 10 Å, as the initial reference, with D1 symmetry. Particles were subjected to per-particle CTF refinement^38^ and Bayesian polishing^46^ before the final refinement. Using standard post-processing in RELION, the reconstructed map was automatically sharpened with a B-factor of −82.96 Å^2^ and masked with a soft-edge solvent mask enclosing 40% of the box in the filament axis direction. The global resolution was estimated based on the gold-standard Fourier shell correlation (FSC) 0.143 criterion between two independently refined half-maps. The local resolution was calculated using RELION. Cryo-EM data collection and 3D reconstruction statistics are shown in Extended Data Table 1.

### Model building, refinement and validation

To aid model building, the post-processed map was locally sharpened using Phenix Autosharpen (from Phenix v1.18.2-3874)^47^ using a high-resolution cut-off of 3.0 Å. An initial model of a single TNKS2 SAM–PARP^G1032W^ protomer was built in Coot^48^ after fitting the TNKS2 PARP domain (PDB code 5NWG, chain IB)^33^ and SAM domain (PDB code 5JRT)^4^ into the central region of the sharpened cryo-EM map where resolution was highest, using UCSF Chimera (v1.14)^49^. A TNKS2 SAM–PARP^G1032W^ filament of ten protomers per protofilament was obtained by applying helical and D1 symmetry to the protomer (equivalent to chain E of the presented model) in UCSF Chimera. The resulting filament model, containing ten protomers in each protofilament (A to J in sense direction, and K to T in antiparallel direction; see Extended Data Fig. 3j), was real-space refined using Phenix Refine (from Phenix v1.18.2-3874)^47^ (maximum of 100 iterations, 5 macrocycles, target bond root-mean-square deviation of 0.01, target angles root-mean-square deviation of 1.0, secondary structure restraints, non-crystallographic symmetry (NCS) detection and refinement, and Ramachandran restraints). Chain E was extracted and subjected to multiple iterations of model building in Coot, symmetrization in UCSF Chimera and real-space refinement in Phenix Refine. The final model was validated using MolProbity^50^, implemented in the Phenix software package. The FSC map versus model plot was calculated using mtriage in Phenix^51^. Model refinement and validation statistics are shown in Extended Data Table 1.

### TNKS2 SAM negative-stain EM and image processing

Wild-type TNKS2 SAM (867–940) was produced recombinantly and purified as previously described^4^. Of purified TNKS2 SAM, 3 µl at 104 μM was applied to the clean side of carbon on a carbon–mica interface, after which the carbon was floated on 2% uranyl acetate. A 400-mesh Cu grid (EMS) was placed on top of the carbon, then picked up with newsprint and laid flat on filter paper to drain the stain. Sixty-seven micrographs were collected on a Tecnai F20 microscope (Thermo Fisher Scientific) equipped with a F416 CMOS detector (TVIPS GmbH), operated at 200 kV with a pixel size of 1.732 Å per pixel.

CTF estimation was performed with GCTF (v1.06)^52^. Particles were manually selected using e2helixboxer.py in EMAN2 (v2.9)^43^, and 9,921 overlapping segments with a box size of 176 pixels were extracted. All further processing steps were carried out in RELION (v3.1.0)^38^. Several rounds of reference-free 2D classification were conducted, and the best class averages were used as templates for reference-based auto-picking. Particles (*n* = 20,131) were subsequently extracted and subjected to 2D classification, resulting in a cleaned dataset of 18,720 particles. A first round of asymmetric 3D refinement was carried out against a featureless cylinder, resulting in a map with a clear D1 symmetry axis. A second round of refinement was then carried out against this map with applied D1 symmetry, yielding a final map that showed clear features of a two-start helix. Projections of the final map were compared with projections of both the SAM domain filament from the SAM–PARP model and the single-stranded TNKS2 SAM domain filament crystal structure (PDB ID 5JRT^4^), low-pass filtered to 20 Å.

### Interface analysis using PDBePISA

Interfaces were characterized using PDBePISA (Proteins, Interfaces, Structures and Assemblies; https://www.ebi.ac.uk/pdbe/pisa/)^21^. Similarity searches for PARP–PARP head-like and tail-like interfaces were performed with the E–R and E–Q chain pairs, respectively, and the following parameters: 70% sequence similarity, multimeric assembly found or not found, head or tail interface found, and any other interface found or not found. Complementary searches for chains not in head-like or tail-like contacts were performed with otherwise identical parameters. All non-tankyrase hits were removed for the subsequent analysis. Domain pairs were superimposed onto the E–R or E–Q PARP–PARP domain pairs using MatchMaker in UCSF Chimera (v1.14)^49^. To compare domain conformations (Extended Data Fig. 5c and Supplementary Table 1), all redundant chains in the set (arising from crystallographic symmetry) were removed; that is, molecules related by crystallographic symmetry were counted only once. In cases in which single domains were modelled as two separate chains, the chains missing from the PDBePISA interface file were added manually. Domains were superimposed onto the PARP domain from chain E of the TNKS2 SAM–PARP filament. The similarity search for the tail interface using the E–Q chain pair missed a number of clearly identifiable tail-like pairs. To include this population of domain pairs, an additional search was performed with the A–A chain pair from PDB 5DCZ (which had the highest Q score in the similarity search with the E–Q chain pair) and results from both searches combined.

To explore whether small-molecule tankyrase inhibitors could determine the D-loop base conformation, we compared the conformations of 239 PARP domains not engaged in a head-like interaction. We reasoned that within this subset, the D-loop would remain unrestrained (whereas a PARP–PARP head interface would stabilize the open D-loop base). An A-site (or dual-site) binder was invariably associated with an open D-loop base. Of the 18 domains with an open D-loop base, 13 featured small-molecule inhibitors occupying the A-site. The remaining five domains with an open D-loop base featured N-site binders, but also electron densities within the A-site, which were often partially modelled as glycerol (Extended Data Fig. 5c and Supplementary Table 1). In the two domains without a bound small-molecule inhibitor, the D-loop base was closed (Supplementary Table 1). Chain B of 3MHJ^31^ (with an N-site binder) displayed alternate D-loop base conformations (Extended Data Fig. 5d), suggesting that the D-loop can sample both conformations in the absence of an A-site binder. Together, these observations support the notion that A-site binders can induce and/or stabilize an open D-loop base. We speculate that an open D-loop base may encourage the PARP–PARP head interaction and thereby support tankyrase polymerization.

### Structure representation and analysis

For TNKS2 SAM–PARP, secondary structure was assigned using the STRIDE algorithm^53^, provided on a web interface (http://webclu.bio.wzw.tum.de/cgi-bin/stride/stridecgi.py)^54^. The output from the web interface was converted to PDB format using http://www.canoz.com/sdh/STRIDEtoPDBsecondarystruct.pl. Figures were generated using UCSF Chimera (v1.14) and ChimeraX (v1.3), developed by the Resource for Biocomputing, Visualization, and Informatics at the University of California, San Francisco, with support from NIH P41-GM103311, R01-GM129325, and the Office of Cyber Infrastructure and Computational Biology, National Institute of Allergy and Infectious Diseases^49,55^. Contacts were defined by atoms with a van der Waals (VDW) overlap of −0.5 Å or greater.

### Luciferase reporter assays

Luciferase reporter assays were performed as previously described^4^, using the TOPFlash reporter containing six TCF/LEF-binding sites, which respond to active β-catenin^56^. HEK293T cells were seeded on white 96-well plates at 26,000 cells per well. Twenty-four hours later, cells were transfected in technical quadruplicate with pLP-dMYC-SD-TNKS2 (16 ng per well), TOPFlash (10 ng per well) and ptkRL (2 ng per well). DNA was filled up to a total amount of 50 ng per well using pDNR-MCS-SA^35^. Cell media were changed for 100 μl Opti-MEM I (Thermo Fisher Scientific/Gibco), and cells were transfected using Lipofectamine 2000 (Thermo Fisher Scientific/Invitrogen) using a DNA:transfectant ratio of 1:3 in Opti-MEM I. Four hours after complex addition, media were changed for DMEM with 0.3% FBS. Twenty hours following media change, cells from two technical replicates were lysed using passive lysis buffer (Promega) and processed for luminometry using the Dual-Luciferase Reporter Assay system (Promega). Plates were read using a POLARstar Omega plate reader (BMG Labtech), using the Omega software (v5.70). Data were analysed using Microsoft Excel for Mac (v16.57) and GraphPad Prism (v9.3.1). Upon background subtraction, ratios of firefly luciferase to Renilla luciferase signals were calculated for each of the two technical replicates. The means of the technical replicates were further analysed as indicated in the figure legends. Cells from the third and fourth technical replicates were lysed in 2× SDS sample buffer and used to monitor the expression levels of TNKS2 by western blotting. Data shown are from at least three independent experiments, as detailed in the figure legends.

### Full-length TNKS2 PARP activity assays

HEK293T cells were seeded on 10-cm dishes at 3.5 × 10^6^ cells per dish. Twenty-four hours later, cells were transfected with a pLP-dMYC-SD empty vector or the indicated TNKS2 constructs (two dishes for WT, K1042A and K1014A and one dish for other constructs) using calcium phosphate. Twenty-four hours post-transfection, cells were scraped in ice-cold PBS and cell pellets snap-frozen in liquid nitrogen. Cell pellets were lysed in 1 ml high-salt RIPA buffer (50 mM HEPES-NaOH (pH 7.5), 750 mM NaCl, 1% Triton X-100, 0.5% sodium deoxycholate, 0.1% SDS, 1 mM DTT, 1 μM PDD000172173 PARG inhibitor (Merck) and protease inhibitors (Pierce protease inhibitor tablets, EDTA-free, Thermo Fisher Scientific)). Cell lysates were briefly sonicated on ice to shear DNA and cleared by centrifugation (20,817*g* for 15 min) at 4 °C. The cleared cell lysates were incubated with 30 μl (50% slurry) of pre-equilibrated anti-MYC magnetic resin (9E10, Thermo Fisher Scientific) for 2 h, rotating at 4 °C. The magnetic resin was washed nine times with 1 ml lysis buffer and three times with 1 ml PARP assay buffer (50 mM HEPES-NaOH (pH 7.5), 150 mM NaCl, 0.01% Triton X-100, 10% glycerol and 1 mM DTT). After the final wash step, each sample was split into two (for incubation with or without NAD^+^), and resin was resuspended in 41 μl of PARP assay buffer to achieve a final volume of 45 μl. The resuspension volume was based on the estimation that half the volume of slurry is made up of resin, and the resin traps roughly half its volume in buffer. Each reaction was incubated with 200 μM NAD^+^, or with H_2_O as control, for 30 min at 30 °C with shaking (800 rpm). The reaction was stopped by adding 25 μl of 4× SDS sample buffer and boiled for 5 min at 100 °C. Samples were analysed by western blotting using pan-ADP-ribose binding reagent (MABE1016, Millipore). Levels of pan-ADP-ribose were quantified using ImageJ software (NIH, v2.3.0/1.53f), which involved background correction (using an empty lane) and normalizing to the levels of TNKS2 variants quantified from the without-NAD^+^ conditions. For quantitation of in vitro PARylation, the portion of the lane corresponding to TNKS2 and above was measured (primarily auto-PARylation, see examples in Supplementary Fig. 1 (1) and (5)). Where background correction gave rise to negative values, these were constrained to zero. Data were expressed relative to WT TNKS2.

### Expression and purification of His_6_-MBP–TNKS2 SAM–PARP variants for biochemical and biophysical assays

His_6_-MBP–TNKS2 SAM–PARP variants were expressed either in *E. coli* or Sf9 insect cells.

Proteins expressed in *E. coli* were prepared as described above for TNKS2 SAM–PARP^G1032W^ (867–1162), except for WT His_6_-MBP–TNKS2 SAM–PARP, for which 1% (w/v) glucose was added to the preculture media to limit leaky expression that resulted in toxicity. Cells were resuspended in a buffer containing 50 mM HEPES-NaOH (pH 7.5), 0.5 M NaCl, 10 mM β-mercaptoethanol and 10% glycerol with cOmplete EDTA-free protease inhibitor cocktail tablets (Roche) (50 ml of lysis buffer and one protease inhibitor tablet added for each litre of culture). Cells were lysed by sonication in a Vibra-Cell sonicator (Sonics & Materials) and centrifuged to remove insoluble cellular debris. The lysate was filtered with a 0.45-μm filter and loaded onto a 5-ml MBPTrap HP affinity column (GE Healthcare/Cytiva). The column was washed with at least 5 CV of wash buffer (as lysis buffer without protease inhibitors). Proteins were eluted with a linear maltose gradient (0–50 mM) in a buffer also containing 50 mM HEPES-NaOH (pH 7.5), 0.5 M NaCl, 10 mM β-mercaptoethanol and 10% glycerol. The protein was dialysed overnight against 50 mM HEPES-NaOH (pH 7.5), 0.5 M NaCl, 2 mM TCEP and 10% glycerol without the addition of TEV protease. Protein samples were flash-frozen in liquid nitrogen and stored at −80 °C.

For NAD^+^-binding assays, the WT, VY903/920WA and G1032W mutant variants of His_6_-MBP–TNKS2 SAM–PARP variants were expressed in Sf9 insect cells as we observed a higher ADP-ribosylation activity and lower basal auto-PARylation levels for proteins produced in insect cells as compared with *E. coli*. We expressed proteins from codon-optimized cDNAs (GenScript) subcloned into the 4C pFastBac vector (Supplementary Table 2). Viral bacmids for the protein expression were generated using Tn7 transposition in chemically competent DH10 MultiBacTurbo *E. coli* cells. Recombinant baculoviruses were generated in Sf9 cells by transfection with purified bacmids using Cellfectin II reagent (cat. no. 10362100, Thermo Scientific). Proteins were expressed in 400 ml of Sf9 cell cultures at a density of 1 × 10^6^ cells per millilitre. Cells were infected with the amplified recombinant baculoviruses and incubated at 27 °C under mild shaking (270 rpm) until the viability dropped below 80% (typically after 96 h). Cells were collected by centrifugation at 250*g* for 10 min at 4 °C and cell pellets were frozen at −80 °C. Cell lysates were prepared as described for proteins expressed in *E. coli*. Filtered lysates were loaded onto a 5-ml HisTrap column (cat. no. 17524802, Cytiva), pre-equilibrated in wash buffer (50 mM HEPES-NaOH (pH 7.5), 0.5 M NaCl, 10 mM β-mercaptoethanol, 20 mM imidazole and 10% glycerol), and the column was washed with 10 CV of wash buffer or until baseline UV absorption at 280 nm was observed. Proteins were eluted using a linear imidazole gradient (20–350 mM) in a buffer containing 50 mM HEPES-NaOH (pH 7.5), 0.5 M NaCl, 10 mM β-mercaptoethanol and 10% glycerol. Fractions containing the fusion protein were pooled and dialysed against a final buffer containing 50 mM HEPES-NaOH (pH 7.5), 0.5 M NaCl, 2 mM TCEP and 10% glycerol. Proteins were concentrated using Vivaspin Turbo concentrators (cat. no. VS15T21, Sartorius) or small-volume (0.5 ml) Pierce Protein Concentrators (cat. no. 88513, Pierce).

### His_6_-MBP–TNKS2 SAM–PARP activity assays

Equal amounts (1 µM) of purified recombinant proteins in a final purification buffer (50 mM HEPES-NaOH (pH 7.5), 500 mM NaCl and 2 mM TCEP and 10% glycerol) were incubated with or without 0.2 mM NAD^+^ for 30 min at 30 °C in a thermoblock (VWR) with mild shaking (180 rpm). A higher concentration of NAD^+^ (0.5 mM) was used for the experiments shown in Extended Data Fig. 1. The reaction was stopped by adding 4× SDS loading buffer. Samples were resolved by SDS–PAGE, and ADP-ribosylation was detected using an anti-pan-ADP-ribose binding reagent (1:10,000 dilution; MABE1016, Millipore). Protein loading was assessed by Ponceau S (cat. no. P7170, Sigma-Aldrich) or Revert 700 total protein stain (cat. no. 926-11021, LI-COR).

### Western blotting

For mammalian-cell based assays, proteins were extracted by boiling cell pellets or resin with immunoprecipitated proteins in SDS sample buffer. Proteins were then resolved by SDS–PAGE and electroblotted onto nitrocellulose membranes. Following blocking in 5% dried skimmed milk powder dissolved in Tris-buffered saline with 0.1% Tween 20 (TBST), membranes were incubated with primary antibodies overnight at 4 °C. Primary antibodies were anti-MYC (1:1,000; 9E10, Abcam), anti-pan-ADP-ribose (immunoprecipitates: 1:4,000, purified proteins: 1:10,000; MABE1016, Millipore), anti-α-tubulin (1:1,000; MA119162, Thermo Fisher) and anti-β-actin (1:1,000; A00702-40, GenScript). Membranes were then washed in TBST and incubated for at least 1 h with appropriate fluorescently conjugated secondary antibodies (immunoprecipitates: 1:15,000, purified proteins 1:30,000; IRDye 680RD/IRDye 800CW, LI-COR). After washing in TBST, bound secondary antibodies were detected using the LI-COR Odyssey imaging system with LI-COR Image Studio software (v5.2.5).

### Mass photometry

#### Instrument setup

Microscope coverslips (High Precision, no. 1.5, 24 × 50 mm, #630-2187, VWR) were cleaned twice, sequentially with Milli-Q water, 100% isopropanol, Milli-Q water. Washed coverslips were dried with compressed air. Reusable silicone CultureWell gaskets (#GBL103250, Sigma-Aldrich) were cleaned sequentially with Milli-Q water, 100% isopropanol, Milli-Q water and dried with compressed air. Cleaned gaskets were placed on the cleaned coverslips and mounted on the sample stage of a Refeyn One^MP^ mass photometer (Refeyn Ltd). All measurements were performed in acquisition buffer containing 50 mM HEPES-NaOH (pH 7.5), 500 mM NaCl and 2 mM TCEP, and filtered through a 0.2-μm filter.

#### Data acquisition

Purified His_6_-MBP–TNKS2 SAM–PARP variants were diluted in acquisition buffer to a concentration of 10 μM and incubated on ice for 30 min. Just before mass photometry measurements, 12 μl of buffer were pipetted into a well and used to identify the focal position for data collection using the autofocus function. Samples were diluted to an intermediate concentration of 250 nM and then immediately to a final concentration of 50 nM in the well (3 μl of 250 nM protein added to the 12-μl drop of buffer), except for the Y920A/G1032W variant, which was diluted to a final concentration of 10 nM because of the high number of binding events in the field of view due its monomeric state. Data acquisition was performed using the Refeyn Acquire^MP^ software (v2.3.1). Mass photometry movies of 6,000 frames were recorded from a 10 × 10 μm instrument field of view at a frame rate of 362.4 Hz, with a frame binning value of 4, resulting in an effective frame rate of 90.6 Hz.

#### Data processing

Data were processed and analysed using Refeyn Discover^MP^ software (v2.3.0) by performing three main steps: (1) background removal, (2) identification of particle landing events on the coverslip acquisition field, and (3) particle fitting to extract maximum contrast. Default parameters were used for all steps. Individual particle contrasts from each individual movie were converted to mass using a contrast-to-mass calibration. Five datasets for each His_6_-MBP–TNKS2 SAM–PARP variant were merged to give a single kernel density estimate curve, using a Gaussian kernel with a fixed bandwidth of 5 kDa.

The minimum between monomer and dimer peaks on the kernel density estimate curve for each variant was used as a cut-off to estimate the proportion of monomeric/oligomeric species in the sample. Three variants (PARP–PARP tail combination, PARP–PARP head–tail combination and SAM/linker–PARP combination [PARP]) gave lower-quality data, characterized by multiple ‘sticky’ binding/unbinding species, low numbers of fitted events and poor definition between peaks. For these variants, the monomer/oligomer cut-off was instead taken from the G1032W variant. These cut-offs were then applied to the five datasets for each variant to calculate the mean percentage of monomeric and >monomeric events, with s.e.m., using Microsoft Excel for Mac (v16.57) and GraphPad Prism (v9.3.1). Note that the quantifications in Fig. 5e and Extended Data Fig. 9b show the percentages of particles that are monomeric or multimeric; this is distinct from the percentages of protein molecules in different assembly states (monomeric or >monomeric). For example, a mass photometry peak corresponding to dimers and of equal height to the monomer peak contains twice as many proteins.

#### Mass calibration

Contrast-to-mass calibration was performed in acquisition buffer. NativeMark unstained protein standard (#LC0725, Thermo Fisher Scientific), which contains proteins in the range of 20–1,200 kDa, was diluted 1:100 in acquisition buffer, and 5 μl were added to a 12-μl drop in a well for measurement. The following masses were used to generate a standard calibration curve in the Discover^MP^ software: 146, 480 and 1,048 kDa.

As polymers will dissociate upon dilution, measurements probably reflect dissociation products. This explains how mutations in the PARP–PARP head interface affect polymerization, although the detected polymeric species are smaller than the shortest polymers that would enable PARP–PARP domain contacts.

### Fluorescence polarization

Fluorescence polarization (FP) experiments were performed with the non-hydrolysable NAD^+^ analogue BAD^22^, labelled at the primary amine of the adenine group with 6-carboxyfluorescein (6-FAM) via a 1,6-diaminohexane linker (synthesized by Jena Bioscience). WT and mutant variants of His_6_-MBP–TNKS2 SAM–PARP protein at increasing concentrations were incubated with 50 nM of BAD in FP buffer (50 mM HEPES-NaOH (pH 7.5), 500 mM NaCl, 2 mM TCEP and 0.01% CHAPS) for 30 min at room temperature in a 96-well half-area plate (cat. no. 675076, Greiner). Fluorescence intensities were read on a BMG Pherastar plate reader (with a 485-nm excitation and two 520-nm emission filters; PHERAstar FSX software (BMG, v5.70 R4)) and background-corrected. FP values were calculated in the MARS data analysis software (BMG, v3.42.105.44). Three separate experiments were performed in technical duplicate, and averages of the technical duplicates were subsequently analysed. To obtain ΔFP values, baseline correction was performed by subtracting the zero-protein well FP value from each of the other FP values. To calculate *K*_d_ values, a non-linear regression was performed using the one-site-specific binding model in GraphPad Prism (v9.3.1). The ΔFP curves for polymerizing proteins probably reflect a combination of NAD^+^ binding and concentration-dependent polymerization; hence, the *K*_d_ values reflect apparent affinities. Note that the assay assumes that the filaments formed by WT His_6_-MBP–TNKS2 SAM–PARP under the experimental conditions are of sufficient length to enable PARP–PARP head interactions. As mass photometry requires substantial dilution, this is difficult to confirm. WT His_6_-MBP–TNKS2 SAM–PARP showed evidence of protein aggregation at the highest concentration points (13 µM and 26 μM). Therefore, the titration of the WT protein was limited to a maximum of 6.5 μM.

### Fluorescence microscopy

HeLa Flp-In T-Rex mCitrine–TNKS2 cell lines were seeded directly onto coverslips (18 × 18 mm) at 6 × 10^4^ cells per coverslip. Thirty-two hours later, cells were treated with 100 ng ml^−1^ doxycycline with or without 1 μM TNKS1/2 inhibitor, Compound 21 (ref. ^57^). Forty-eight hours post-seeding, coverslips were incubated with CellMask (Thermo Fisher Scientific) for 10 min at 37 °C. Coverslips were then washed twice with PBS, and cells were fixed with 4% formaldehyde for 10 min at 37 °C. Coverslips were washed three times with PBS, and cells were stained with Hoechst 33258 (5 μg ml^−1^; Merck/Sigma) for 30 min at room temperature. Coverslips were next washed three times with PBS and mounted onto slides using VECTASHIELD (VECTOR Laboratories). Cells were imaged 24–48 h post-fixation using a Zeiss Axio Observer Z1 Marianas microscope. Z-stacks were imported into ImageJ/FIJI software (NIH, v2.3.0/1.53f) and used to generate maximum intensity projections for analysis. At least two coverslips were analysed per condition. CellProfiler (Broad Institute, v3.1.9) was used to quantitate the number of puncta per cell and the size (area) of each individual punctum from image projections. The analysis pipeline was created to (1) identify cytoplasm using CellMask staining, (2) identify objects within this area using the IdentifyPrimaryObjects function, and (3) measure the number of puncta per cell and their size (area) in pixels. The average number of puncta per cell and the average size of puncta were calculated for three independent experiments, with a minimum of 150 cells quantified per condition in each experiment (Supplementary Table 4). A one-way ANOVA with a Tukey test for multiple comparisons was performed in GraphPad Prism (v9.3.1) (Supplementary Table 5). Images used in figures are maximum intensity projections generated from Z-stacks. A uniform exposure adjustment across all panels in Photoshop (Adobe, v2021) was used to enhance features in the figures.

### Differential scanning fluorimetry

Thermal stability of the His_6_-MBP–TNKS2 SAM–PARP WT and mutant proteins was assessed using differential scanning fluorimetry^58,59^. Five µM of WT or mutant protein in purification buffer (20 mM HEPES-NaOH (pH 7.5), 500 mM NaCl and 2 mM TCEP) were mixed with 5x SYPRO Orange (Invitrogen; relative to 5,000 × stock of undisclosed concentration), and 20 μl of each setup were transferred onto a 96-well PCR plate. Melting curves were obtained on a QuantStudio 6 Flex PCR machine (Thermo Fisher Scientific) with QuantStudio real-time PCR software (v1.7.1). Temperature increases were linear from 22 °C to 95 °C with 0.5 °C per 15-s increments. Fluorescence intensity was measured in real time using the ROX channel. Average fluorescence intensities from three parallel experiments for each protein were normalized (0–100%), smoothed and plotted against the temperature using GraphPad Prism (v9.3.1). The melting temperatures (*T*_m_; defined as 50% of the maximum fluorescence intensity) were obtained directly from the resulting melting curves and showed good agreement with those obtained from the peaks of the first derivative curves.

### Phylogenetic analyses

Multiple sequence alignments were generated with Clustal Omega^60^ using the web services from the EMBL-EBI^61^ (version January 2021). UniProt entry names for human ARTD family members are PARP1_HUMAN, PARP2_HUMAN, PARP3_HUMAN, PARP4_HUMAN, PAR15_HUMAN, PAR14_HUMAN, PARP9_HUMAN, PAR10_HUMAN, PAR11_HUMAN, PAR12_HUMAN, ZCCHV_HUMAN, PARPT_HUMAN, PAR16_HUMAN, PARP8_HUMAN and PARP6_HUMAN. NCBI accession numbers for TNKS1 orthologues are NP_003738.2 (*Homo sapiens*), NP_780300.2 (*Mus musculus*), NP_989671.1 (*Gallus gallus*), XP_035410112.1 (*Cygnus atratus*), XP_019389515.1 (*Crocodylus porosus*), XP_037752450.1 (*Chelonia mydas*), XP_026528659.1 (*Notechis scutatus*), XP_018099067.1 (*Xenopus laevis*), XP_005451454 (*Oreochromis niloticus*), ENSDART00000111694.5 (*Danio rerio*), XP_005171802.1 (*D. rerio*), XP_014351007.1 (*Latimeria chalumnae*), XP_036384182.1 (*Megalops cyprinoides*) and XP_032873798.1 (*Amblyraja radiata*). NCBI accession numbers for TNKS2 orthologues are NP_079511.1 (*H. sapiens*), NP_001157107.1 (*M. musculus*), NP_989672.1 (*G. gallus*), XP_035404219.1 (*C. atratus*), XP_019411065.1 (*C. porosus*), XP_007059469.2 (*C. mydas*), XP_026526558.1 (*N. scutatus*), XP_018082988.1 (*X. laevis*), XP_005471626.1 (*O. niloticus*), XP_006006371.1 (*L. chalumnae*), XP_036385759.1 (*M. cyprinoides*), XP_032889463.1 (*A. radiata*) and XP_020371197.1 (*Rhincodon typus*). Two tankyrase paralogues appear to emerge in cartilaginous fish (Chondrichthyes). NCBI accession numbers for all other tankyrase orthologues are XP_032806710.1 (*Petromyzon marinus*), XP_002121662.3 (*Ciona intestinalis*), XP_019641281.1 (*Branchiostoma belcheri*), XP_789260.4 (*Strongylocentrotus purpuratus*), XP_022094330.1 (*Acanthaster planci*), NP_651410.1 (*Drosophila melanogaster*), XP_321116.5 (*Anopheles gambiae*), XP_032783964.1 (*Daphnia magna*), XP_023333893.1 (*Eurytemora affinis*), XP_029842287.1 (*Ixodes scapularis*), GBM18725.1 (*Araneus ventricosus*), XP_022240762.1 (*Limulus polyphemus*), CAD5118347.1 (*Dimorphilus gyrociliatus*), XP_005099438.1 (*Aplysia californica*), XP_022340347.1 (*Crassostrea virginica*), KRZ50196.1 (*Trichinella nativa*), KHN72016.1 (*Toxocara canis*), CDS23197.1 (*Echinococcus granulosus*), TNN12026.1 (*Schistosoma japonicum*), XP_012563232.1 (*Hydra vulgaris*) and XP_019848937.1 (*Amphimedon queenslandica*). Alignments were visualized and coloured in Jalview (v2.10.5)^62^. For Extended Data Fig. 6, the stretches of sequences that were not aligning with the reference sequence (human TNKS2) were deleted for clarity. Percentage identity across tankyrase paralogues and orthologues, as per the alignment omitting the non-tankyrase ARTD family members, was mapped onto TNKS2 SAM–PARP using UCSF Chimera (v1.14)^49^, using a linear red-to-white colour gradient from 100% to 90% and white for identity lower than 90%.

### Generation of figures

Publication figures were generated using Adobe Photoshop and Illustrator (v2021).

### Reporting summary

Further information on research design is available in the Nature Portfolio Reporting Summary linked to this article.

## Online content

Any methods, additional references, Nature Portfolio reporting summaries, source data, extended data, supplementary information, acknowledgements, peer review information; details of author contributions and competing interests; and statements of data and code availability are available at 10.1038/s41586-022-05449-8.

### Supplementary information

Supplementary Information This file contains the western blot source images (Supplementary Fig. 1), the legend for Supplementary Table 1, Supplementary Tables 2–5, and the legend for Supplementary Video 1.

Reporting Summary

Supplementary Table 1

Supplementary Video 1

## Figures and Tables

**Extended Data Table 1:**
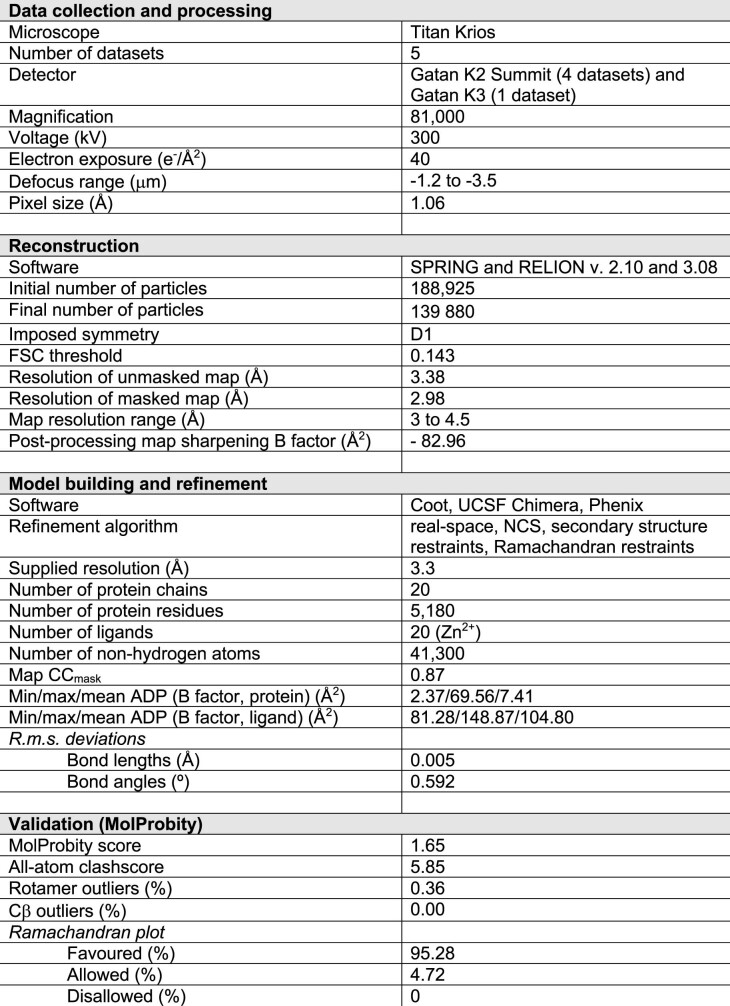
Cryo-EM data collection, 3D reconstruction and model refinement & validation statistics

## Data Availability

Cryo-EM maps and raw EM movie datasets of TNKS2 SAM–PARP^G1032W^ were deposited at the EM Data Resource with accession codes EMD-15520 and EMPIAR-11227, respectively. Structural coordinates of the refined model were deposited at the PDB with the accession code 8ALY.
