# Supplementary material for: Structural basis of tankyrase activation by polymerization
Source: Nature. 2022 Nov 23;612(7938):162–9. doi: 10.1038/s41586-022-05449-8 (PMC9712121; doi:10.1038/s41586-022-05449-8)
Supplement: Supplementary file 1 — This file contains the western blot source images (Supplementary Fig. 1), the legend for Supplementary Table 1, Supplementary Tables 2–5, and the legend for Supplementary Video 1. [file 41586_2022_5449_MOESM1_ESM.docx]

**Supplementary information**

**Structural basis of tankyrase activation by polymerisation**

Nisha Pillay^1,2^*, Laura Mariotti^1,2^*, Mariola Zaleska^1,2^, Oviya Inian^1,2^, Matthew Jessop^1,2^, Sam Hibbs^1,2^, Ambroise Desfosses^3^, Paul C.R. Hopkins^1,2^, Catherine M. Templeton^1,2^, Fabienne Beuron^1^, Edward P. Morris^1^, Sebastian Guettler^1,2,✉^

^1^ Division of Structural Biology, The Institute of Cancer Research (ICR), London, United Kingdom

^2^ Division of Cancer Biology, The Institute of Cancer Research (ICR), London, United Kingdom

^3^ Institut de Biologie Structurale (IBS), University Grenoble Alpes, CEA, CNRS, Grenoble, France

* These authors contributed equally.

**Table of Contents**

**Supplementary Fig. 1 (1):** Source images of Fig. 4…….……………………………….……...3

**Supplementary Fig. 1 (2):** Source images of Extended Data Fig. 1……………….………….4

**Supplementary Fig. 1 (3):** Source images of Extended Data Fig. 7g-h….…………………...5

**Supplementary Fig. 1 (4):** Source images of Extended Data Fig. 7i-k……….……………….6

**Supplementary Fig. 1 (5):** Source images of Extended Data Fig. 8a-b………….…………...7

**Supplementary Fig. 1 (6):** Source images of Extended Data Fig. 8c-d…………….…………8

**Supplementary Fig. 1 (7):** Source images of Extended Data Fig. 8e-f……….………...…….9

**Supplementary Fig. 1 (8):** Source images of Extended Data Fig. 8g-h……….…………….10

**Supplementary Fig. 1 (9):** Source images of Extended Data Fig. 8i……….………………..11

**Supplementary Table 1:** PISA domain interface and conformational analysis of TNKS/TNKS2 PARP domain structures available in the PDB…………………………………12

**Supplementary Table 2:** Plasmids used in this study………………….……………………...12

**Supplementary Table 3:** Combination mutant variants described in this study not explicitly named after the point mutation………………………………………………………………….…13

**Supplementary Table 4:** Number of cells analysed by fluorescence microscopy……….....13

**Supplementary Table 5:** Statistical analysis of fluorescence microscopy…………………..14

**Supplementary Video 1:** Comparison of mass photometry movies for TNKS2 SAM-PARP variants…………………………………………………………………………………………..…..14

**
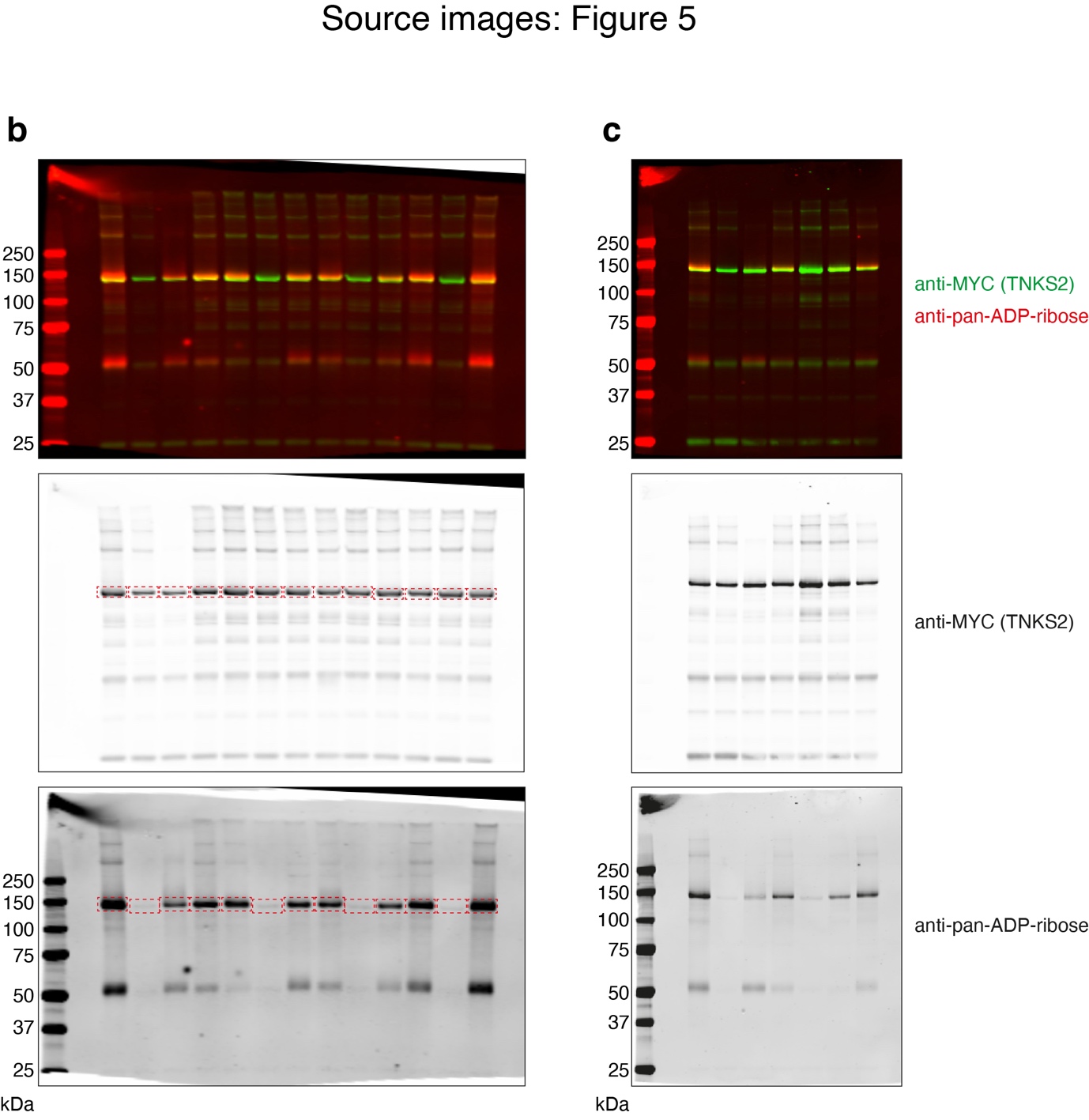
**

**Supplementary Fig. 1 (1): Source images of Fig. 5.**

Boxes with red dashed lines illustrate example areas used for quantification, in this case for the “endogenous” ADP-ribosylation signals.

**
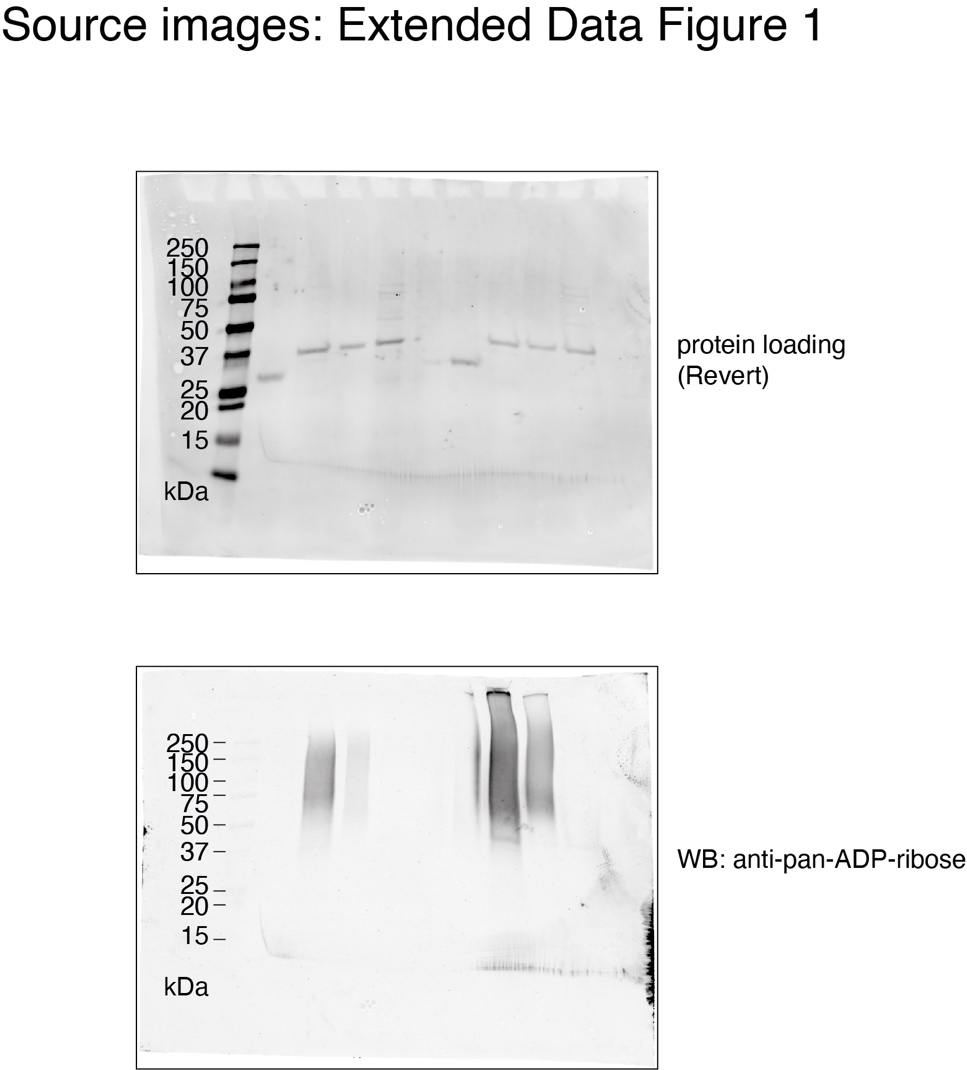
**

**Supplementary Fig. 1 (2): Source images of Extended Data Fig. 1.**

**
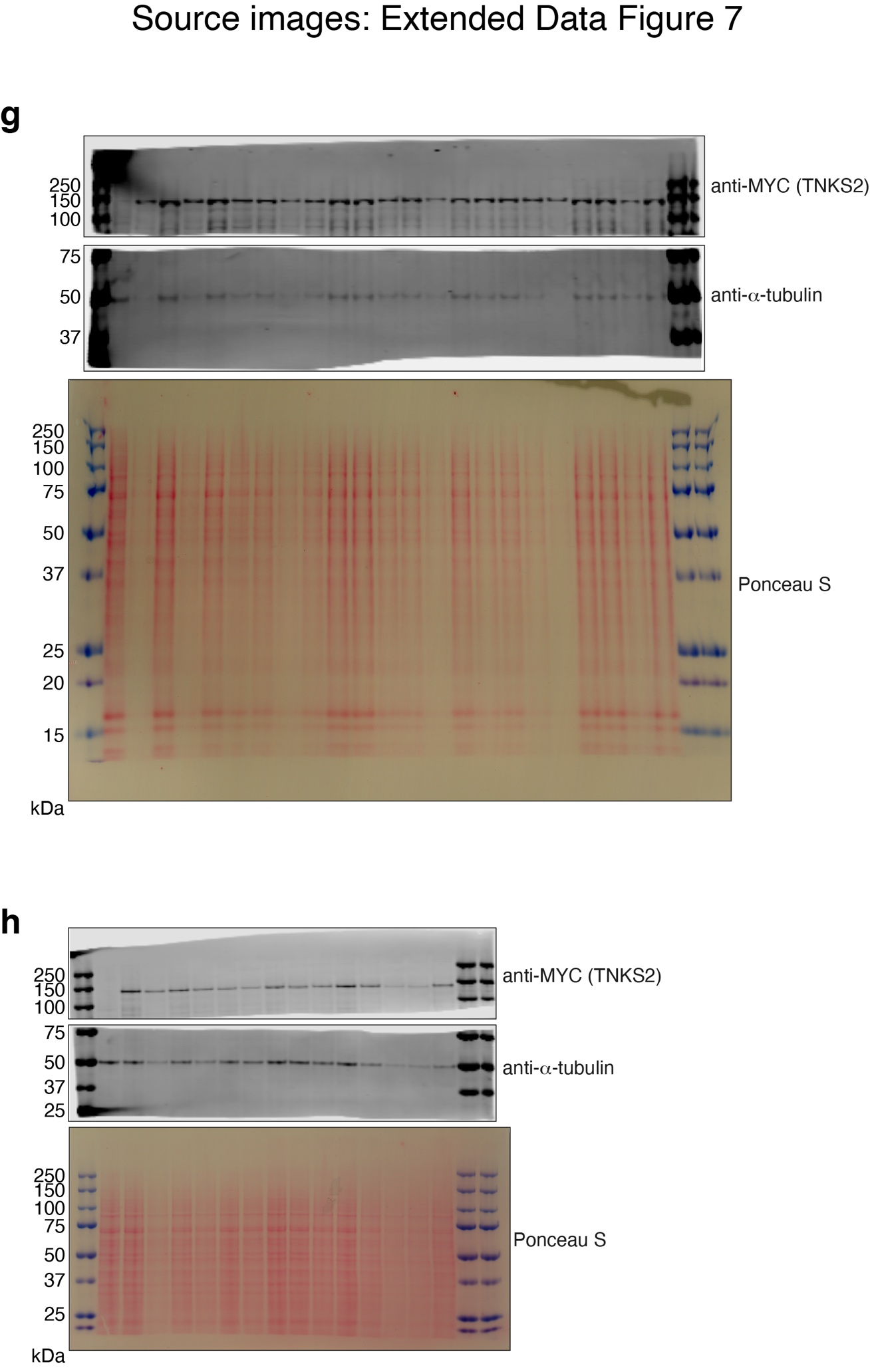
**

**Supplementary Fig. 1 (3): Source images of Extended Data Fig. 7g-h.**

**
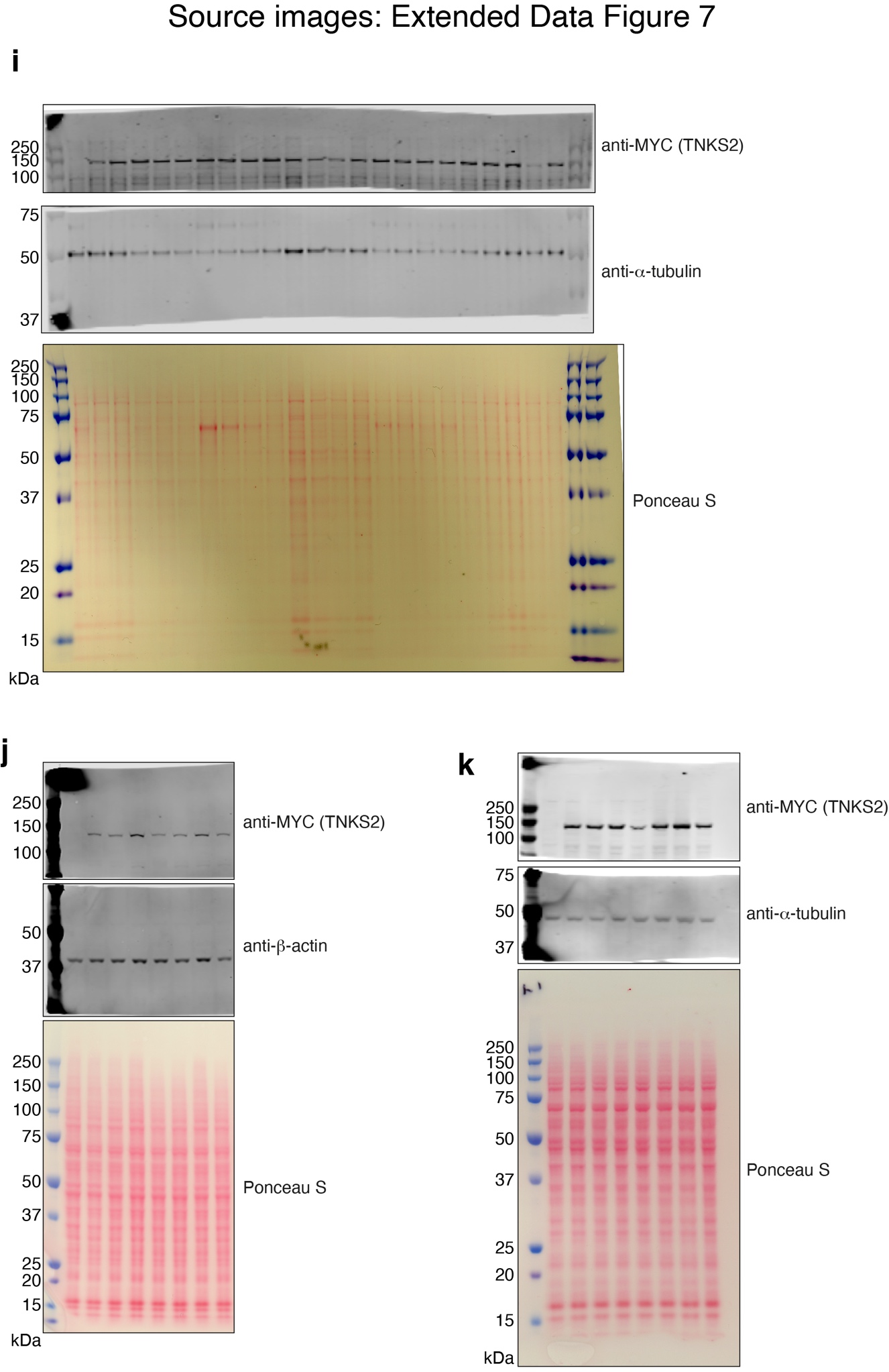
**

**Supplementary Fig. 1 (4): Source images of Extended Data Fig. 7i-k.**

**
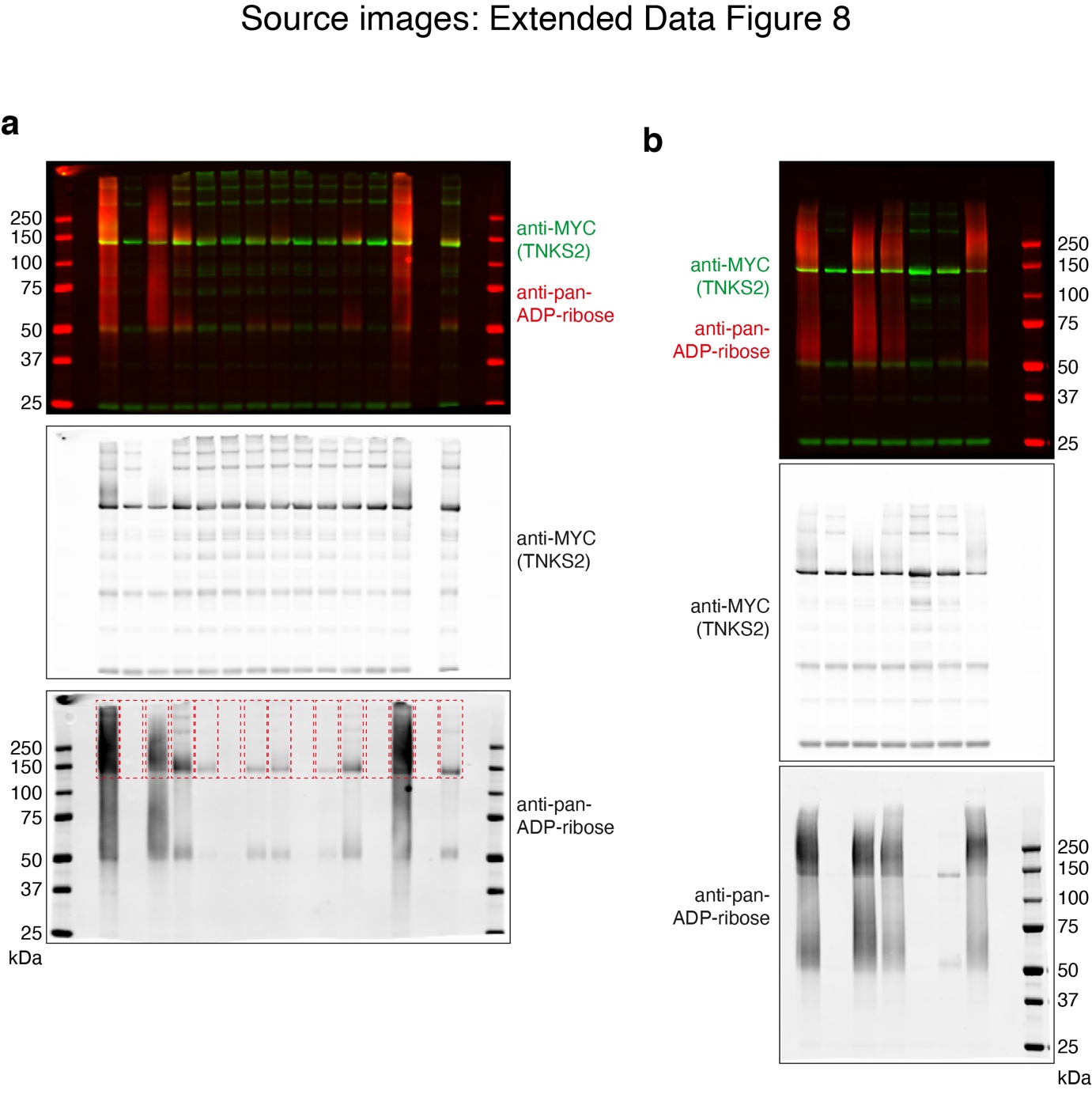
**

**Supplementary Fig. 1 (5): Source images of Extended Data Fig. 8a-b.**

Boxes with red dashed lines illustrate example areas used for quantification, in this case for the ADP-ribosylation signals after *in vitro* ADP-ribosylation.

**
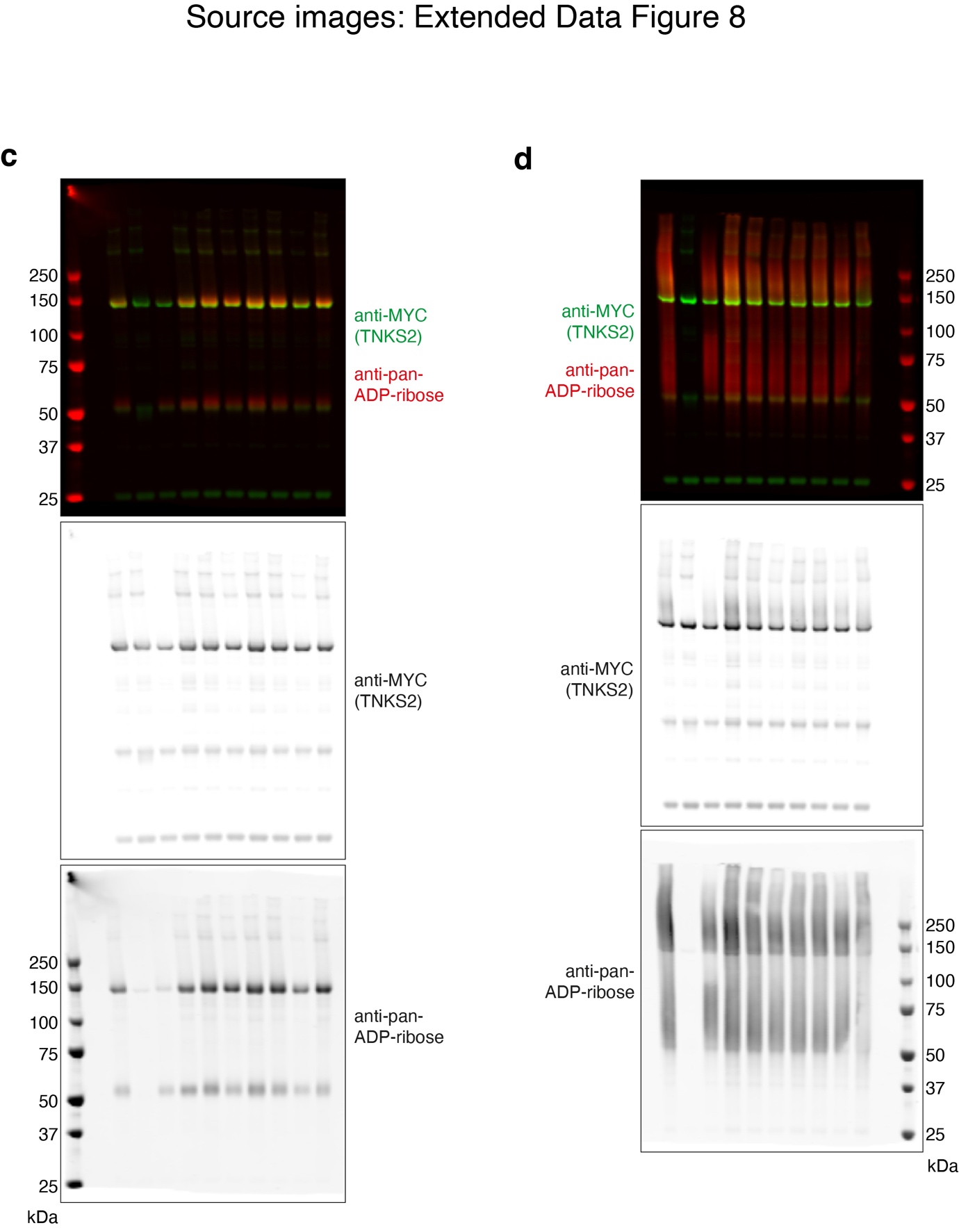
**

**Supplementary Fig. 1 (6): Source images of Extended Data Fig. 8c-d.**

**
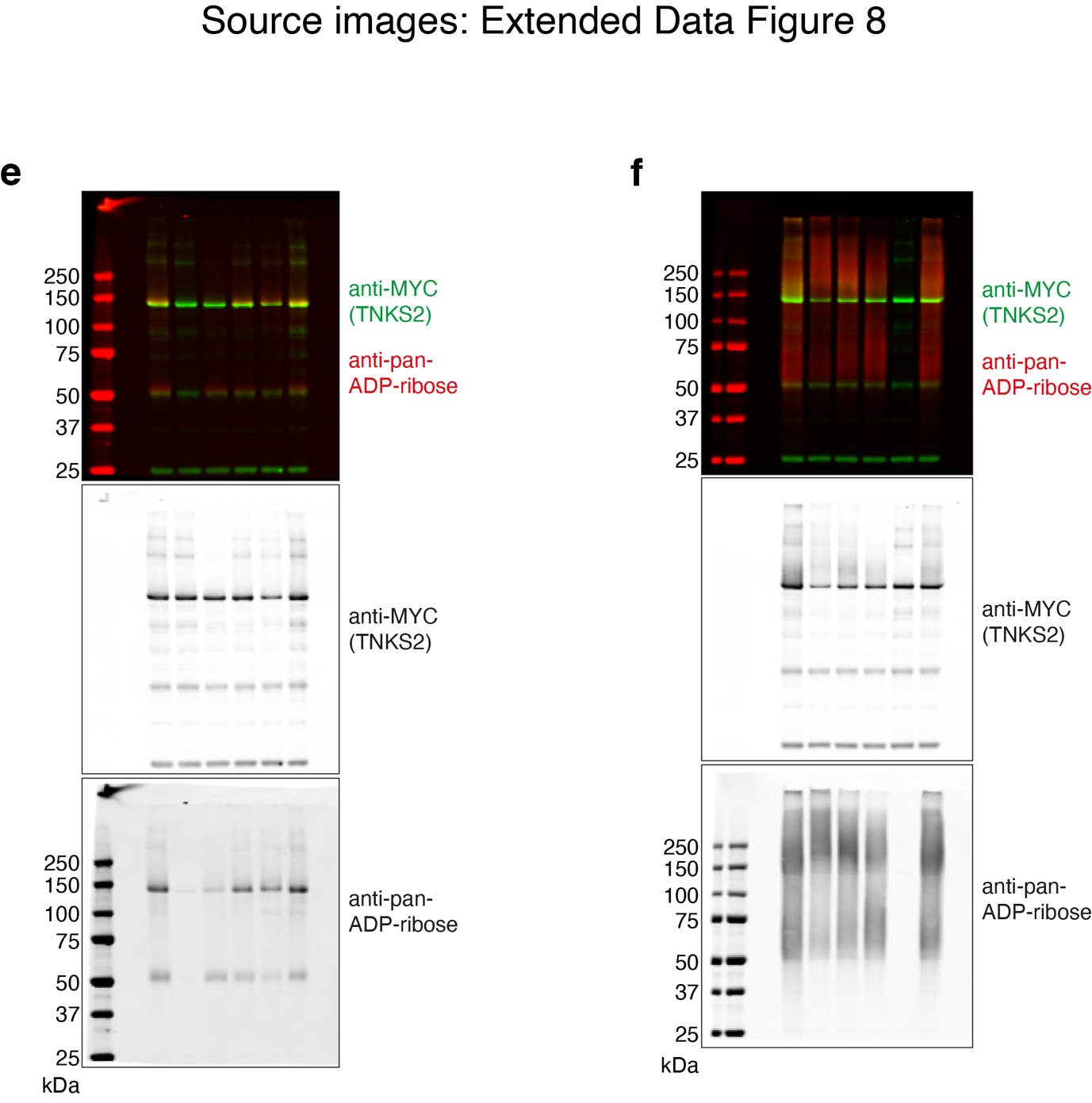
**

**Supplementary Fig. 1 (7): Source images of Extended Data Fig. 8e-f.**

**
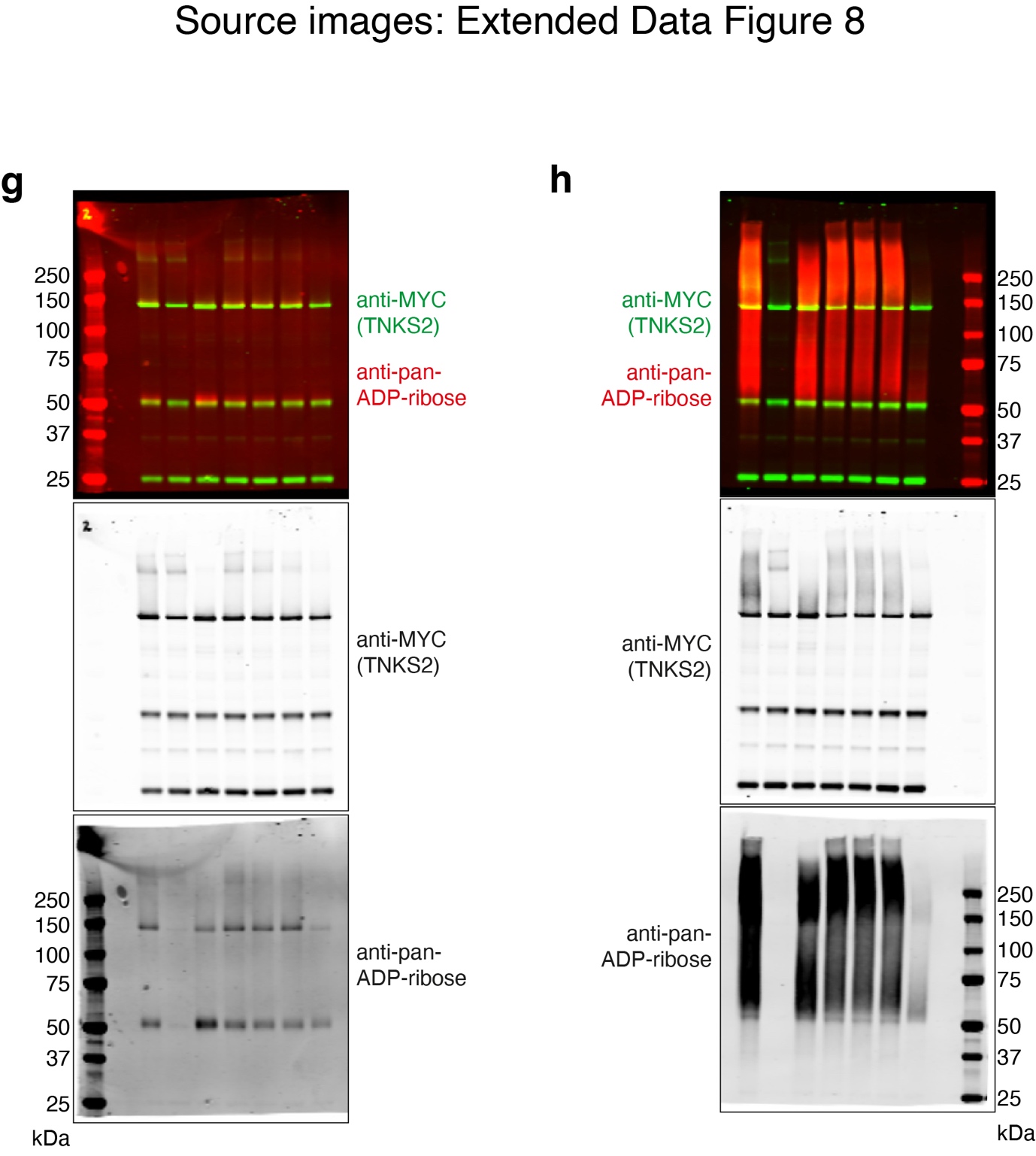
 Supplementary Fig. 1 (8): Source images of Extended Data Fig. 8g-h.**

**
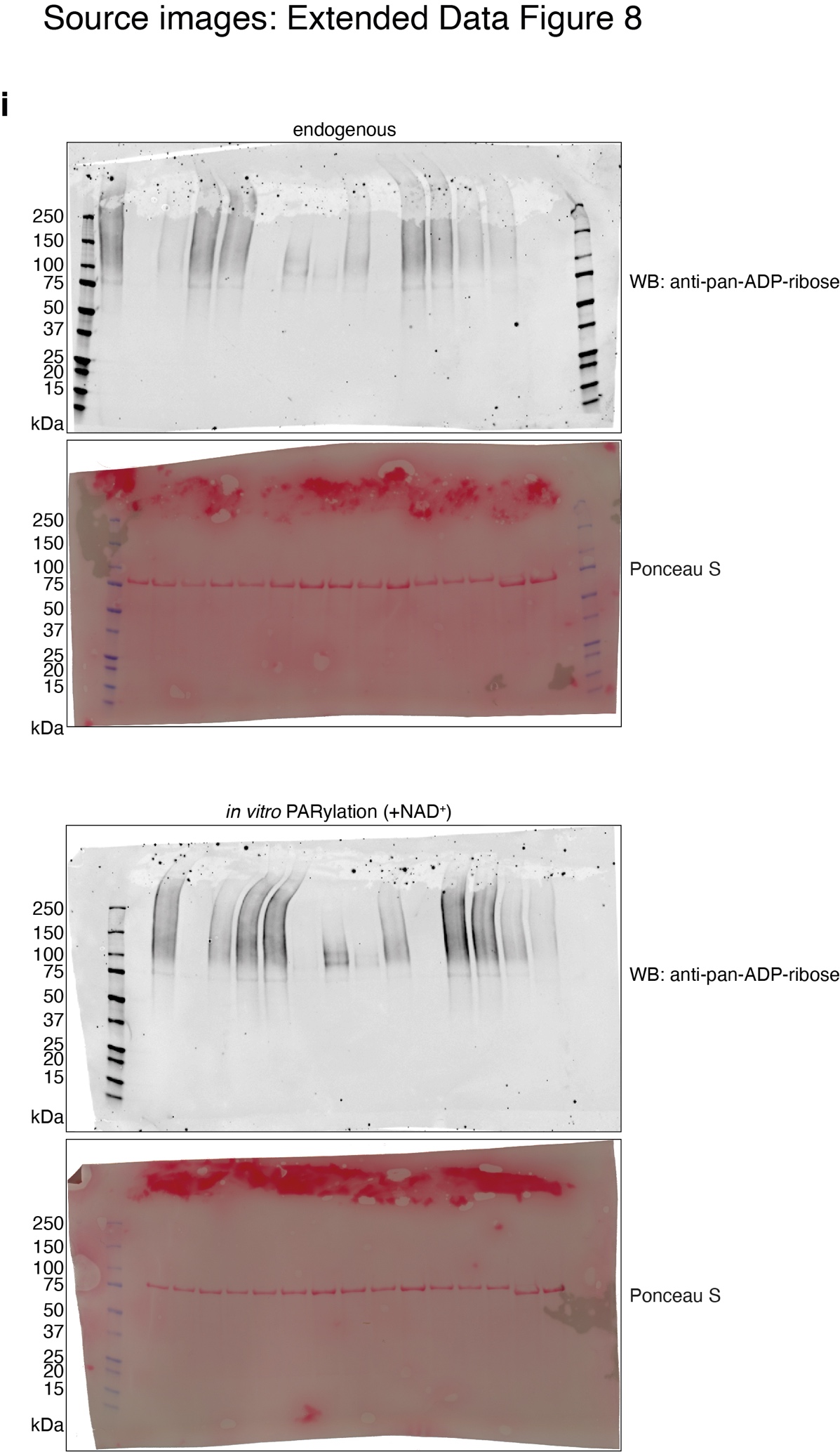
**

**Supplementary Fig. 1 (9): Source images of Extended Data Fig. 8i.**

**Supplementary Table 1 (Excel spreadsheet): PISA domain interface and conformational analysis of TNKS/TNKS2 PARP domain structures available in the PDB.**

Sheet 1, domains in head-like contact; sheet 2, domains not in head-like contact; sheet 3, domains in tail-like contact; sheet 4, domains not in tail-like contact; sheet 5, side-by-side list of PDB codes. For domains not in head-like contact, the presence of an N-, A- or dual-site-binding small-molecule inhibitor is indicated. Engagement of the A-site correlates with an open D-loop base. Electron density maps for domains displaying an open D-loop base but no small-molecule within the A-site display densities in the A-site, which in some cases are modelled as glycerol but extend further. Without small-molecule inhibitors bound, the D-loop base is closed. The adoption of alternate D-loop base conformations (open, closed) in the same PARP domain chain (3MHJ, chain B)^31^ suggests that in the absence of an A-site binder and a head-like PARP:PARP interaction, the D-loop can sample both conformations.

**Supplementary Table 2: Plasmids used in this study.**

| **plasmid name** | **species** | **accession no.** | **sites** | **references/information** |
| --- | --- | --- | --- | --- |
| **bacterial expression constructs** | | | | |
| pET-His_6_-MBP-Asn_10_-TEV (1C)-TNKS2(867-1162) (SAM-PARP) | human | NM_025235 | LIC v1 | 1 |
| pET-His_6_-MBP-Asn_10_-TEV (1C)-TNKS2(867-940) (SAM) | human | NM_025235 | LIC v1 | 1, 2 |
| pET-His_6_-MBP-Asn_10_-TEV (1C)-TNKS(952-1162) (PARP) | human | NM_025235 | LIC v1 | 1 |
| **mammalian expression constructs** | | | | |
| pLP-dMyc SD | - | - | - | 3 |
| pLP-dMyc SD-TNKS2 | human | NM_025235 | *AscI-PacI & loxP* | 2, 3 |
| M50 Super 8x TOPFlash | - | - | *-* | 4 |
| ptkRL | - | - | *-* | 5 |
| pDNR-MCS SA | - | - | *-* | 3 |
| pcDNA5-FRT/TO-mCitrine-TNKS2 | human | NM_025235 |  | 6 |
| **baculovirus/insect cell expression constructs** | | | | |
| pFastBac-His_6_-MBP-Asn_10_-TEV (4C)-TNKS2(867-1162) (SAM-PARP) | human | NM_025235, codon-optimised for *E. coli* | LIC v1 | 1 |

^1^ The empty vectors were a gift from Dr. Scott Gradia (UC Berkeley) via Addgene (Addgene plasmids # 29654 and 30116)

^2^ reference^4^

^3^ reference^34^

^4^ M50 Super 8x TOPFlash, obtained via Addgene, was a gift from Randall Moon (Addgene plasmid # 12456)^35^

^5^ ptkRL, originally from Promega, was a gift from Dr. Richard Treisman (Francis Crick Institute, London, UK).

^6^ pcDNA5-FRT/TO was a gift from Dr. Anne-Claude Gingras (Lunenfeld-Tanenbaum Research Institute, Toronto, Canada).

**Supplementary Table 3: Combination mutant variants described in this study not explicitly named after the point mutation.**

| **Mutant variant** | **Point mutations** |
| --- | --- |
| PARP:PARP head comb. | P1120G, H1117A, E1046A, R1143E, H1011A |
| PARP:PARP tail comb. | H1021A, H1023A, M1028A, R1100A, K1105A |
| PARP:PARP head+tail comb. | P1120G, H1117A, E1046A, R1143E, H1011A, H1021A, H1023A, M1028A, R1100A, K1105A |
| SAM/linker:PARP comb. [SAM] | Q880A, N884A, L901A, G939R, L940A |
| SAM/linker:PARP comb. [PARP] | I954A, I956A, K999A, K1003A, W1006A, Y1148A, E1150A |

**Supplementary Table 4: Number of cells analysed by fluorescence microscopy.**

| number of cells analysed (without TNKSi) | | | | | | |
| --- | --- | --- | --- | --- | --- | --- |
| replicate | WT | G1032W | VY903/920WA | PARP:PARP  head comb. | PARP:PARP tail comb. | PARP:PARP head+tail comb. |
| 1 | 266 | 220 | 178 | 422 | 413 | 725 |
| 2 | 411 | 450 | 262 | 468 | 461 | 531 |
| 3 | 384 | 390 | 343 | 508 | 561 | 346 |
| number of cells analysed (with TNKSi) | | | | | | |
| replicate | WT | G1032W | VY903/920WA | PARP:PARP  head comb. | PARP:PARP tail comb. | PARP:PARP head+tail comb. |
| 1 | 200 | 239 | 128 | 508 | 442 | 657 |
| 2 | 339 | 471 | 271 | 496 | 521 | 483 |
| 3 | 498 | 357 | 256 | 467 | 488 | 256 |

**Supplementary Table 5: Statistical analysis of fluorescence microscopy.**

*P*-values for one-way ANOVA with Tukey’s test for multiple comparisons are shown. Data are from three independent experiments.

| **Comparison** | ***P*-value** |
| --- | --- |
| Average number of puncta per cell | |
| WT vs. G1032W | 0.0189 |
| WT vs. VY903/920WA | 0.9779 |
| WT vs. PARP:PARP head comb. | 0.4030 |
| WT vs. PARP:PARP tail comb. | 0.9946 |
| WT vs. PARP:PARP head+tail comb. | 0.0256 |
| G1032W vs. VY903/920WA | 0.0058 |
| G1032W vs. PARP:PARP head comb. | 0.4199 |
| G1032W vs. PARP:PARP tail comb. | 0.0450 |
| G1032W vs. PARP:PARP head+tail comb. | >0.9999 |
| VY903/920WA vs. PARP:PARP head comb. | 0.1488 |
| VY903/920WA vs. PARP:PARP tail comb. | 0.8205 |
| VY903/920WA vs. PARP:PARP head+tail comb. | 0.0079 |
| PARP:PARP head comb. vs. PARP:PARP tail comb. | 0.6873 |
| PARP:PARP head comb. vs. PARP:PARP head+tail comb. | 0.5165 |
| PARP:PARP tail comb. vs. PARP:PARP head+tail comb. | 0.0609 |
| Average size of puncta (in pixels) | |
| WT vs. G1032W | 0.0022 |
| WT vs. VY903/920WA | 0.9998 |
| WT vs. PARP:PARP head comb. | <0.0001 |
| WT vs. PARP:PARP tail comb. | 0.3913 |
| WT vs. PARP:PARP head+tail comb. | 0.2377 |
| G1032W vs. VY903/920WA | 0.0033 |
| G1032W vs. PARP:PARP head comb. | 0.0853 |
| G1032W vs. PARP:PARP tail comb. | 0.0610 |
| G1032W vs. PARP:PARP head+tail comb. | 0.1115 |
| VY903/920WA vs. PARP:PARP head comb. | <0.0001 |
| VY903/920WA vs. PARP:PARP tail comb. | 0.5319 |
| VY903/920WA vs. PARP:PARP head+tail comb. | 0.3430 |
| PARP:PARP head comb. vs. PARP:PARP tail comb. | 0.0005 |
| PARP:PARP head comb. vs. PARP:PARP head+tail comb. | 0.0008 |

**Supplementary Video 1 (mov video file): Comparison of mass photometry movies for TNKS2 SAM-PARP variants.**

Representative crops of mass photometry ratiometric movies for the indicated TNKS2 SAM-PARP variants. Binding events appear as spots with a black centre, whereas unbinding events appear as spots with a white centre. Species marked with asterisks show a higher tendency of molecules to repeatedly bind and unbind to and from the glass surface (see Methods for details).
