# Supplementary material for: Structural basis of tankyrase activation by polymerization
Source: Nature. 2022 Nov 23;612(7938):162–9. doi: 10.1038/s41586-022-05449-8 (PMC9712121; doi:10.1038/s41586-022-05449-8)
Supplement: Supplementary file 2 — Reporting Summary [file 41586_2022_5449_MOESM2_ESM.pdf]

nature portfolio

Corresponding author(s): Sebastian Guettler

Last updated by author(s): 2022/09/21

Reporting Summary

Nature Portfolio wishes to improve the reproducibility of the work that we publish. This form provides structure for consistency and transparency in reporting. For further information on Nature Portfolio policies, see our Editorial Policies and the Editorial Policy Checklist.

Statistics

For all statistical analyses, confirm that the following items are present in the figure legend, table legend, main text, or Methods section.

n/a	Confirmed	
☐	☒	The exact sample size (*n*) for each experimental group/condition, given as a discrete number and unit of measurement
☐	☒	A statement on whether measurements were taken from distinct samples or whether the same sample was measured repeatedly
☐	☒	The statistical test(s) used AND whether they are one- or two-sided<br>*Only common tests should be described solely by name; describe more complex techniques in the Methods section.*
☒	☐	A description of all covariates tested
☒	☐	A description of any assumptions or corrections, such as tests of normality and adjustment for multiple comparisons
☐	☒	A full description of the statistical parameters including central tendency (e.g. means) or other basic estimates (e.g. regression coefficient) AND variation (e.g. standard deviation) or associated estimates of uncertainty (e.g. confidence intervals)
☐	☒	For null hypothesis testing, the test statistic (e.g. *F*, *t*, *r*) with confidence intervals, effect sizes, degrees of freedom and *P* value noted<br>*Give P values as exact values whenever suitable.*
☒	☐	For Bayesian analysis, information on the choice of priors and Markov chain Monte Carlo settings
☒	☐	For hierarchical and complex designs, identification of the appropriate level for tests and full reporting of outcomes
☒	☐	Estimates of effect sizes (e.g. Cohen's *d*, Pearson's *r*), indicating how they were calculated

Our web collection on statistics for biologists contains articles on many of the points above.

Software and code

Policy information about availability of computer code

Data collection	EM: EPU (v. 1.14.0.54)<br>Mass photometry: Refeyn Acquire (v. 2.3.1)<br>FP: PHERAstar FSX (BMG, v. 5.70 R4)<br>DSF: QuantStudio real-time PCR software (v. 1.7.1)<br>Luciferase reporters: Omega software (v. 5.70)<br>Western blots: LI-COR Image Studio (v. 5.2.5)
Data analysis	EM: MotionCor2, RELION (v. 2.10, 3.08 and 3.1.0), CTFFIND4, GCTF (v. 1.06), Bshow (from Bsoft v. 1.9.5), e2align2d.py (from EMAN2 v. 2.31), HELIXPLORER (v. March 2018), SPRING (v. 0.86.1661), EMAN2 (v. 2.31 and 2.9)<br>Structure model building, refinement, validation, analysis and representation: Coot (v. 0.9), PHENIX (v. 1.18.2-3874), STRIDE algorithm (Heinig et al., 2004), MolProbity (implemented in PHENIX), UCSF Chimera (v. 1.14) and ChimeraX (v. 1.3)<br>Western blot quantification: ImageJ (v. 2.3.0/1.53f)<br>Analysis of fluorescence micrographs: ImageJ/FIJI (NIH, v. 2.3.0/1.53f), CellProfiler™ (Broad Institute, v. 3.1.9)<br>Mass Photometry: Refeyn Discover (v. 2.3.0)<br>FP: MARS data analysis software (BMG, v. 3.42.105.44)<br>Alignments: Clustal Omega (v. January 2021), Jalview (v. 2.10.5)<br>Plotting and statistical analysis: Microsoft Excel for Mac (v. 16.57), GraphPad Prism (v. 9.3.1)<br>Figure preparation: Adobe Photoshop and Illustrator (v. 2021)

For manuscripts utilizing custom algorithms or software that are central to the research but not yet described in published literature, software must be made available to editors and reviewers. We strongly encourage code deposition in a community repository (e.g. GitHub). See the Nature Portfolio guidelines for submitting code & software for further information.

Data

Policy information about availability of data

All manuscripts must include a data availability statement. This statement should provide the following information, where applicable:

- Accession codes, unique identifiers, or web links for publicly available datasets
- A description of any restrictions on data availability
- For clinical datasets or third party data, please ensure that the statement adheres to our policy

Cryo-EM maps and raw EM movie datasets of TNKS2 SAM-PARP G1032W were deposited at the EM Data Resource with accession codes EMD-15520 and EMPIAR-11227, respectively. Structural coordinates of the refined model were deposited at the Protein Data Bank (PDB) with accession code 8ALY.

Human research participants

Policy information about studies involving human research participants and Sex and Gender in Research.

Reporting on sex and gender	n/a
Population characteristics	n/a
Recruitment	n/a
Ethics oversight	n/a

Note that full information on the approval of the study protocol must also be provided in the manuscript.

Field-specific reporting

Please select the one below that is the best fit for your research. If you are not sure, read the appropriate sections before making your selection.

☒ Life sciences ☐ Behavioural & social sciences ☐ Ecological, evolutionary & environmental sciences

For a reference copy of the document with all sections, see nature.com/documents/nr-reporting-summary-flat.pdf

Life sciences study design

All studies must disclose on these points even when the disclosure is negative.

Sample size	No statistical methods were used to predetermine sample sizes. Experiments were repeated as indicated to establish reproducibility.
Data exclusions	No data were excluded from analyses.
Replication	Biochemical, biophysical and cell-based experiments were repeated independently at least three times, as indicated, with similar results.
Randomization	This study does not include experiments that require randomisation.
Blinding	This study does not include experiments that require blinding. Potential bias from prior knowledge of samples is limited.

Reporting for specific materials, systems and methods

We require information from authors about some types of materials, experimental systems and methods used in many studies. Here, indicate whether each material, system or method listed is relevant to your study. If you are not sure if a list item applies to your research, read the appropriate section before selecting a response.

Materials & experimental systems

n/a	Involved in the study	
☐	☒	Antibodies
☐	☒	Eukaryotic cell lines
☒	☐	Palaeontology and archaeology
☒	☐	Animals and other organisms
☒	☐	Clinical data
☒	☐	Dual use research of concern

Methods

n/a	Involved in the study	
☒	☐	ChIP-seq
☒	☐	Flow cytometry
☒	☐	MRI-based neuroimaging

Antibodies

Antibodies used

anti-MYC (9E10, ab206486, Abcam), anti-pan-ADP-ribose (MABE1016, Millipore, LOT# 3223347), anti-alpha-tubulin (TU-01, MA119162, ThermoFisher, LOT# 531288), anti-beta-actin (2D1D10, A00702, GenScript), Secondary anti-mouse/rabbit (IRDye 680RD/ IRDye 800CW, LI-COR, LOT#: anti-mouse-680, D00311-03; anti-mouse-800, D00115-03; anti-rabbit-680, D00115-06; anti-rabbit-800, D00115-06)

Validation

All antibodies used are commercially available as validated reagents. Specificity was established by Western blotting using non-transfected cells as controls, where applicable, and by the molecular weight of the respective target.

Eukaryotic cell lines

Policy information about cell lines and Sex and Gender in Research

Cell line source(s)

HEK293T: Professor Chris Lord, ICR, London, UK
HeLa Flp-In™ T-Rex™: Professor Stephen Taylor, University of Manchester, UK

Authentication

Cell lines used were authenticated at source. No further authentication was undertaken.

Mycoplasma contamination

negative

Commonly misidentified lines
(See ICLAC register)

No commonly misidentified cell lines were used.
